# Targeting Tumor-Associated Macrophages to Reshape the Immuno-Mechanical Landscape: Molecular Mechanisms and Therapeutic Strategies

**DOI:** 10.7150/ijbs.132476

**Published:** 2026-05-11

**Authors:** Guanghui Liu, Yang Yu, Zichen Guo, Lijuan Liu, Wujin Chen, Changgang Sun

**Affiliations:** 1 College of First Clinical Medicine, Shandong University of Traditional Chinese Medicine, Jinan, Shandong, 250000, China.; 2 Faculty of Chinese Medicine and State Key Laboratory of Mechanism and Quality of Chinese Medicine, Macau University of Science and Technology, Macau, 999078, China.; 3 Department of Oncology, Weifang Traditional Chinese Hospital, Weifang, Shandong, 261000, China.; 4 The Affiliated People's Hospital of Fujian University of Traditional Chinese Medicine, Fuzhou, Fujian, 350122, China.; 5 College of Traditional Chinese Medicine, Shandong Second Medical University, Weifang, Shandong, 261000, China.

**Keywords:** tumor-associated macrophages (TAMs), mechanical microenvironment, extracellular matrix remodeling, tumor softening, therapeutic strategies

## Abstract

The progression and therapeutic resistance of solid tumors are profoundly influenced by the mechanical microenvironment, in which extracellular matrix stiffening, elevated interstitial pressure, and aberrant mechanotransductive signaling constitute critical physical barriers. Tumor-associated macrophages (TAMs) occupy a central position in this process. They not only act as active architects that remodel the matrix and exacerbate fibrosis, but their phenotypes and functions are also reciprocally regulated by the mechanical microenvironment, thereby forming a self-reinforcing malignant loop. Accordingly, targeting TAMs to mechanically soften tumors has emerged as an important therapeutic strategy, encompassing TAMs depletion, reprogramming, inhibition of TAM-mediated the extracellular matrix (ECM) modification, and disruption of mechanosensing pathways. In addition, mechanical immunoengineering and combination therapeutic strategies provide new tools for modulating the tumor mechanical-immune microenvironment. This review systematically examines the bidirectional regulatory mechanisms of TAMs within the mechanical microenvironment and the corresponding therapeutic strategies, and highlights that overcoming spatiotemporal heterogeneity and developing precision intervention paradigms are key to achieving future clinical translation.

## 1. Introduction

The initiation and progression of tumors are not determined solely by intrinsic genetic alterations within tumor cells, but are instead rooted in a highly abnormal tumor microenvironment (TME). This microenvironment comprises multiple cellular components, the extracellular matrix (ECM), vascular and lymphatic networks, as well as a range of physical and mechanical signals^1^. In recent years, research has gradually expanded its focus from biochemical signals to mechanical signals, leading to the proposal of the concept of the tumor mechanical microenvironment^2^. This concept emphasizes that ECM composition and crosslinking, overall tissue stiffness, solid stress, interstitial fluid pressure (IFP), and the permeability and perfusion status of blood and lymphatic vessels together constitute the mechanical basis of tumor growth and evolution^3^, ^4^. Across multiple solid tumors, high collagen deposition and fibrosis, increased tissue stiffness and interstitial pressure, and structurally abnormal and leaky vascular networks have all been demonstrated to be closely associated with enhanced tumor aggressiveness, increased metastatic risk, and poor prognosis. These findings suggest that the mechanical microenvironment itself has become a key dimension for assessing tumor malignancy^5^, ^6^.

An abnormal mechanical microenvironment is not merely a passive consequence of tumor progression, but can actively promote tumor progression and attenuate therapeutic responses through multiple levels of mechanisms^7^. On the one hand, increased matrix stiffness and altered mechanical tension can, via mechanotransduction pathways involving integrins, focal adhesion kinase (FAK), and yes-associated protein (YAP)/ (transcriptional coactivator with PDZ-binding motif) TAZ, accelerate epithelial-mesenchymal transition, maintenance of stemness, and enhancement of invasive capacity in tumor cells, thereby driving local invasion and distant metastasis^8^. On the other hand, dense fibrosis and elevated IFP together form dual physical and functional barriers that compress blood vessels, reduce perfusion, and impede the delivery of chemotherapeutic agents, targeted therapies, and immune cells (particularly CD8^+^ T cells) to the tumor core^9^. In addition, hypoxia and metabolic dysregulation caused by abnormal vasculature and ECM remodeling can further activate immunosuppressive pathways^10^, leading a substantial proportion of patients to develop primary or acquired resistance to immunotherapies such as immune checkpoint inhibitors (ICIs).

Within this complex network, tumor-associated macrophages (TAMs) occupy a critical hub position linking the immune microenvironment and the mechanical microenvironment^11^, ^12^. TAMs are among the most abundant populations of tumor-infiltrating immune cells, and their high-density infiltration is strongly associated with poor patient prognosis, metastasis, and resistance to chemotherapy and radiotherapy^13^, ^14^. Single-cell omics and spatial transcriptomic studies have shown that typical M2-like or immunosuppressive TAMs highly express vascular endothelial growth factor (VEGF), and transforming growth factor-β (TGF-β), interleukin-10 (IL-10), arginase 1 (ARG1), as well as various matrix metalloproteinases (MMPs) and cathepsins, thereby simultaneously driving angiogenesis, suppressing effector T-cell function, and remodeling the ECM^15^-^17^. Recent studies further indicate that fibrotic and hypoxic regions in multiple solid tumors are often enriched with M2-like or lineage-specific TAMs; these cells closely co-localize with cancer-associated fibroblasts (CAFs) and, through the secretion of factors such as MMPs, TGF-β, and C-C motif chemokine ligand 2 (CCL2)/ C-C motif chemokine ligand 5 (CCL5), induce collagen deposition and crosslinking, increase matrix stiffness, and maintain a malignant mechanobiological ecosystem characterized by immune exclusion and high fibrosis^18^, ^19^. Collectively, this evidence indicates that TAMs not only support tumors through immune pathways but also directly participate in the establishment and reinforcement of an abnormal mechanical microenvironment.

On the other hand, TAMs themselves act as integrative responders to mechanical and metabolic signals, and their recruitment, polarization, and functions are profoundly regulated by the mechanical properties of the microenvironment^20^, ^21^. Hypoxia, lactate accumulation, cell-matrix adhesion, and fluid shear stress within the TME collectively shape a highly heterogeneous TAM population^22^, ^23^. Among them, M2-like immunosuppressive TAMs tend to accumulate in regions with high stiffness and high fibrosis that are distant from functional blood vessels^24^. Within these specific mechanobiological niches, TAMs exhibit enhanced transcriptional programs associated with tissue repair and pro-fibrotic activity, thereby further exacerbating ECM remodeling and immunosuppression^15^. Recent reviews have pointed out a close causal relationship between TAM polarization states and multiple physical attributes of the TME, including tissue stiffness, matrix density, and perfusion status^25^. The reciprocal shaping of TAMs by the microenvironment and the remodeling of the microenvironment by TAMs are intricately intertwined, forming a highly dynamic, plastic, yet difficult-to-spontaneously-reverse malignant positive feedback loop^13^.

Despite some progress having been made, there remain several gaps in the current understanding of the interplay between TAMs and the tumor mechanical microenvironment. First, existing studies tend to investigate TAM-mediated immunoregulation and tumor mechanical microenvironment remodeling as relatively independent processes, lacking an integrated theoretical framework that systematically connects immune function with biomechanical evolution. Second, the dynamic and stage-dependent interactions between TAMs and the tumor mechanical microenvironment during tumor progression have not yet been sufficiently elucidated, with most studies focusing on static features and thus failing to capture the temporal characteristics of this bidirectional regulatory system. Third, although both TAM-targeted therapeutic strategies and stroma-modulating approaches have demonstrated certain potential, they have not yet been effectively integrated into a systematic therapeutic framework with clear mechanistic guidance, particularly in overcoming mechano-immunological resistance. These limitations suggest that it is necessary to adopt a more unified and dynamic perspective to re-examine and integrate the TAM-tumor mechanical microenvironment axis, in order to promote the coordinated advancement of mechanistic understanding and therapeutic strategy design.

Therefore, viewing TAMs as a key hub connecting immune networks and the mechanical microenvironment, and reinterpreting their roles in tumor progression and therapeutic responses from a mechanical perspective, may provide new theoretical foundations and potential intervention windows for the development of combined mechanics-immunity intervention strategies. This article systematically elucidates the dual roles of TAMs in both actively shaping the tumor mechanical microenvironment and sensitively responding to mechanical signals within it, and further explores potential approaches and associated challenges in modulating the tumor mechanical microenvironment by targeting TAMs.

## 2. TAMs as architects of the tumor mechanical microenvironment

### 2.1 ECM remodeling and modification

**Figure 1:**
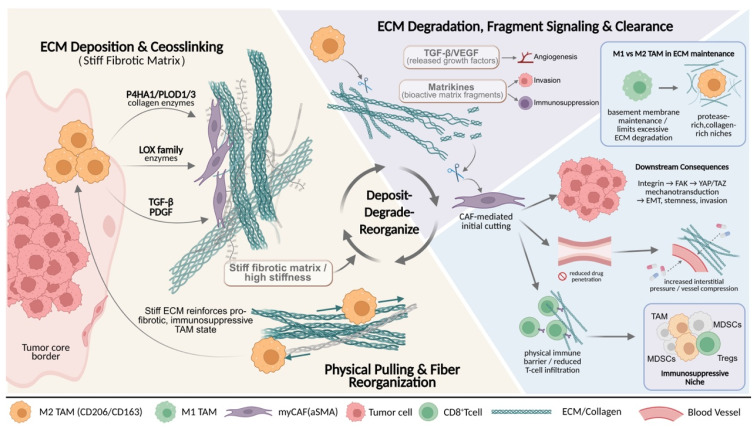
TAM-driven ECM remodeling shapes a stiff, immunosuppressive mechanical niche in solid tumors. Schematic illustration of TAMs-mediated ECM remodeling within the TME. TAMs promote collagen deposition and crosslinking through profibrotic enzymes and cytokines, leading to matrix stiffening and increased interstitial pressure. In parallel, TAMs cooperate with CAFs to orchestrate localized ECM degradation and fiber reorganization, releasing bioactive matrix fragments and growth factors. These dynamic deposit-degrade-reorganize processes generate a mechanically heterogeneous ECM that enhances tumor cell invasion, restricts immune cell infiltration and drug penetration, and favors the accumulation of immunosuppressive myeloid and lymphoid populations. The stiffened ECM further reinforces profibrotic and immunosuppressive TAM states, forming a self-amplifying mechanical-immune feedback loop that drives tumor progression.

Within the tumor mechanical microenvironment, the ECM is not only a physical scaffold supporting tumor cell proliferation and migration, but also a key determinant that integrates biochemical and biomechanical signals and governs tissue stiffness and topological architecture^26^, ^27^. In this process, TAMs play a central processing role. On the one hand, they drive collagen deposition and crosslinking, leading to the formation of a high-stiffness fibrotic matrix^20^, ^25^. On the other hand, through proteolysis and physical traction, they enable localized ECM degradation and fiber rearrangement^28^, ^29^. This continuous dynamic process of deposition, degradation, and reorganization is often carried out cooperatively by TAMs and stromal cells such as CAFs^19^, ^30^, together shaping an ECM microenvironment with highly heterogeneous composition and mechanical properties, thereby influencing tumor invasion patterns, immune cell infiltration, and the efficiency of drug delivery^5^, ^18^ (**Fig. 1**).

ECM remodeling, as a core event in tumor progression, mainly comprises four categories of processes: re-deposition of ECM components and quantity, chemical modifications such as crosslinking, proteolytic degradation, and physical rearrangement of fibers. These changes collectively determine the three-dimensional architecture, stiffness, and degradability of the ECM^31^, ^32^. With respect to ECM synthesis and modification, studies have confirmed that TAMs are an important cellular source of collagen synthesis and maturation^33^. In tumor tissues with high TAM infiltration and in transcriptomic analyses, upregulated expression of multiple ECM components or their regulatory factors (such as type I and type III collagen, fibronectin, and proteoglycans), as well as enzymes that promote collagen crosslinking, can be detected^5^, ^11^, ^34^. In breast cancer and colorectal cancer models, TAMs highly express key enzymes involved in collagen maturation, including Prolyl 4-Hydroxylase Subunit Alpha 1 and PLOD1/3, and initiate collagen biosynthesis through TGF-β-dependent programs, thereby further enhancing stromal fibrosis and tissue stiffness^35^, ^36^. Meanwhile, factors such as TGF-β and platelet-derived growth factor (PDGF) secreted by M2-like TAMs can activate CAFs and induce their differentiation into myofibroblast-like CAFs (myCAFs) with a high ECM-synthetic phenotype^18^, ^37^, ^38^. myCAFs abundantly secrete type I, VI, and XIV collagen, fibronectin, and proteoglycans^6^. In addition, this CAF subset highly expresses ECM signature genes such as COL1A2, POSTN, and SPP1, and spatially co-localizes with TAMs, forming ECM synthesis-centered processing units that continuously reinforce matrix crosslinking and densification^38^, ^39^. Notably, such a stiffened matrix, on the one hand, promotes epithelial-mesenchymal transition and maintenance of stemness through mechanotransduction pathways involving integrins, FAK, and YAP/TAZ^35^, ^40^(6,35,40). On the other hand, by increasing interstitial tissue pressure and compressing blood vessels, it restricts immune cell infiltration and drug delivery^24^. Therefore, ECM stiffening is regarded as an important physical basis driving malignant tumor progression and immune exclusion^36^. More importantly, the stiffened ECM can in turn regulate TAMs, promoting their polarization toward pro-fibrotic and immunosuppressive phenotypes, thereby forming a self-reinforcing malignant cycle.

With respect to ECM degradation, TAM-mediated proteolysis is not a simple destructive process, but rather a finely regulated, stage-specific and stepwise process^28^, ^41^. Multiple studies have shown that M2-like TAMs are an important source of various MMPs, such as MMP 2, MMP 9, and MMP 14, as well as other proteolytic enzymes^15^, ^19^. These enzymes not only directly cleave structural proteins including collagen, laminin, and fibronectin, but also release growth factors stored within the ECM, such as TGFβ and VEGF, and generate bioactive matrix fragments^42^. This further amplifies pro-angiogenic, pro-invasive, and immunosuppressive signals, thereby endowing ECM degradation with dual functions of structural remodeling and signal regulation^18^, ^34^. The contribution of TAMs to ECM degradation is not unidirectional, but functionally complementary with stromal components such as CAFs^18^. Quantitative collagen degradation models have shown that, in multiple solid tumors, the initial cleavage of collagen fibers is primarily mediated by MMPs secreted by CAFs, whereas TAMs cooperate with CAFs to handle the subsequent uptake and lysosomal degradation of collagen fragments^43^. During this process, TAMs highly express multiple ECM receptors and endocytic receptors, among which the mannose receptor (CD206) is particularly critical for the uptake of collagen fragments^44^, ^45^. In tumor models using CD206-deficient mice, collagen uptake by TAMs is significantly impaired, indicating that this receptor is a core molecule mediating TAM-driven ECM degradation^28^, ^46^. Notably, the function of TAMs in ECM degradation exhibits a clear phenotypic bias. M2-like TAMs, marked by CD206 and CD163, are enriched in regions with high collagen content and high proteolytic enzyme activity, whereas M1-like TAMs with antigen-presenting and cytotoxic potential tend to maintain basement membrane integrity and limit excessive degradation^14^, ^29^, ^47^. This phenotype-function association suggests that reprogramming or selectively depleting M2-like TAMs may represent a potential strategy to attenuate the formation of pathological ECM networks^48^.

The ECM collagen network remodeled by TAMs not only constitutes a functional physical barrier, hindering effector T cells and drugs from penetrating into the tumor core, but also shapes an immunosuppressive niche by altering ECM contextual signals and mechanical properties^49^, ^50^. Single-cell and spatial multi-omics analyses show that SPP1^+^ or CD206^+^ M2-like TAMs are highly co-localized with αSMA^+^ CAFs at the tumor boundary and in collagen-enriched regions^51^, ^52^. The two synergistically drive ECM fibrosis and the formation of immune barrier structures, limiting effector T-cell infiltration into the interior of tumors^18^, ^25^. Among them, CAFs mainly dominate matrix densification by secreting large amounts of ECM proteins and regulating the MMP/TIMP balance^53^, ^54^. TAMs are more inclined to synthesize specific ECM components, secrete degradative enzymes, and regulate mechanical signaling, thereby finely controlling ECM plasticity and spatial organizational patterns^18^, ^25^. Together, they constitute a continuous and dynamic ECM processing system. On this basis, abnormal ECM can also remodel the activation threshold and migratory behavior of T cells through receptor signaling such as integrins, and selectively provide anchoring sites and survival signals for immunosuppressive cells such as TAMs, myeloid-derived suppressor cells (MDSCs), and regulatory T cells (Tregs), ultimately jointly promoting an immune cold tumor phenotype and leading to limited efficacy of ICIs^5^, ^55^, ^56^.

### 2.2 Collagen architecture and biomechanical signal guidance

In the construction of the tumor mechanical microenvironment, the function of TAMs goes beyond simply synthesizing or clearing ECM components, and the more central role lies in serving as an organizer of fibrous structures. Through cytoskeleton-mediated active pulling and rearrangement, TAMs can reshape the orientation, spatial connectivity, and tension distribution of collagen fibers^57^, ^58^. This pulling transforms an originally relatively isotropic matrix network into a physical barrier that is mechanically favorable for tumor cell invasion, but at the same time hinders immune cell infiltration^59^, ^60^.

Histological and advanced imaging studies provide intuitive evidence for the close association between TAMs and specific fiber configurations. In multiple solid tumors such as breast cancer and ovarian cancer, regions with high-density TAM infiltration, especially the tumor invasive front, often show characteristic collagen structural changes^51^, ^58^. Specifically, collagen fibers become shorter and thinner, and their arrangement shows a trend toward high parallelism and alignment^59^, ^61^. For example, a study comparing canine and human breast cancer showed that the degree of TAM infiltration is negatively correlated with the average length of collagen fibers, and positively correlated with the alignment of fiber arrangement^58^. This suggests that TAMs not only participate in matrix degradation, but also dominate the systematic rearrangement of fiber architecture, transforming an originally randomly interwoven reticular ECM into an orderly arranged linear structure^34^, ^62^. Such collagen bundles arranged radially along the tumor edge or perpendicular to the boundary have been confirmed to be significantly associated with stronger tumor invasiveness, higher metastatic risk, and a T-cell exclusion phenotype, and thus have become an important morphological indicator for evaluating patient prognosis^63^.

The molecular mechanism by which TAMs achieve ECM fiber rearrangement is rooted in their unique cytoskeletal architecture and mechanosensing capacity. Studies show that when TAMs synergistically carry out ECM degradation and reorganization, they rely on the precise coordination of actin stress fibers, invadopodia, and the intermediate filament network^64^, ^65^. Among these, the intermediate filament network formed by vimentin is crucial for maintaining the functional balance between stress fibers and MT1-MMP-rich invadopodia^66^. As a mechanical scaffold, it can ensure the spatiotemporal coordination of ECM endocytosis, lysosomal degradation, and local export of degradative enzymes^67^, ^68^. Under this mechanism, TAMs first anchor and locally soften collagen fibers through invadopodia, and then apply directional mechanical pulling force via contractile stress fibers, thereby straightening the remaining collagen fiber bundles and realigning them along the direction of cell migration^19^, ^69^. The latest three-dimensional tumor organoid-macrophage co-culture model has intuitively reproduced this process. M2-like TAMs can pull and comb collagen fibers along the migration path of tumor cells, leading to local fiber straightening and alignment. Meanwhile, surrounding fibers that are not directly acted upon maintain their curved conformation, forming a microenvironment with obvious topological partitioning, thereby opening up a directionally migratory channel with low mechanical resistance for tumor cells^62^.

This complex fibrotic process is not an isolated action of TAMs, but rather the result of their precise coordination with other cellular components within the TME. Spatial transcriptomic analyses have revealed that, in triple-negative breast cancer, regions enriched with myCAFs, characterized by high expression of contractile proteins and strong synthetic capacity, are typically associated with denser and more highly aligned collagen bundles^70^-^72^. These regions also correspond to the invasive front, where tumor cells and M2-like TAMs co-accumulate^73^, ^74^. In contrast, areas enriched with immunomodulatory CAFs (iCAFs) exhibit a relatively looser matrix architecture, which is more permissive for the recruitment of various immunosuppressive cell populations^75^. Within this specific ecological niche, TAMs are frequently positioned within the interstices or termini of collagen bundles. By secreting factors such as MMP-9 and lysyl oxidase (LOX), these TAMs not only locally modify the ECM to reduce its resistance to tensile forces, but also enhance the contractile activity of adjacent myCAFs^34^, ^53^. Acting in concert along the same directional axis, TAMs and myCAFs cooperatively exert mechanical forces that efficiently weave large-scale collagenous microstructures with high structural uniformity^38^, ^76^. Emerging evidence suggests that, beyond the aforementioned biochemical paracrine circuits, direct mechanical crosstalk between TAMs and myCAFs may exist in the form of a “cell-matrix-cell” signaling axis. However, the in situ quantitative dissection of such intercellular mechanical interactions remains technically challenging and continues to be an active area of investigation^18^, ^38^.

Given that both cell types anchor to the same ECM network via integrin-mediated adhesions, contractile forces generated by one cell population can be transmitted over long distances along collagen fibers to the other, thereby establishing a mechanically coupled unit. For instance, myCAFs are capable of extensively depositing and crosslinking nascent collagen, thereby constructing a stiff and anisotropic fibrillar scaffold^77^, ^78^; TAMs, in contrast, exhibit pronounced myosin-dependent contractility and possess refined mechanosensory structures, such as invasive protrusions and stress fibers, enabling them to exert directional traction forces on the same ECM fibers via integrins including αvβ3 and α5β1^79^.

This process not only promotes collagen fiber straightening and realignment, but also generates tensile stress along the fibers^80^. Such forces can be directly sensed by adjacent myCAFs through their focal adhesions and integrin-dependent mechanotransduction machinery, thereby further reinforcing their contractile activity and matrix-producing programs^81^. Similarly, perivascular TAMs, such as Tie2⁺ TAMs, through their interactions with endothelial cells, can guide the reorganization of the perivascular basement membrane and collagen fibers along the vascular axis, thereby establishing a structural pattern that facilitates tumor cell migration along vessel walls^82^, ^83^. This ECM scaffold, cooperatively constructed by multiple cell types, provides a structural basis for the directional distribution of IFP and the local amplification of mechanical signaling^84^, ^85^.

**Figure 2:**
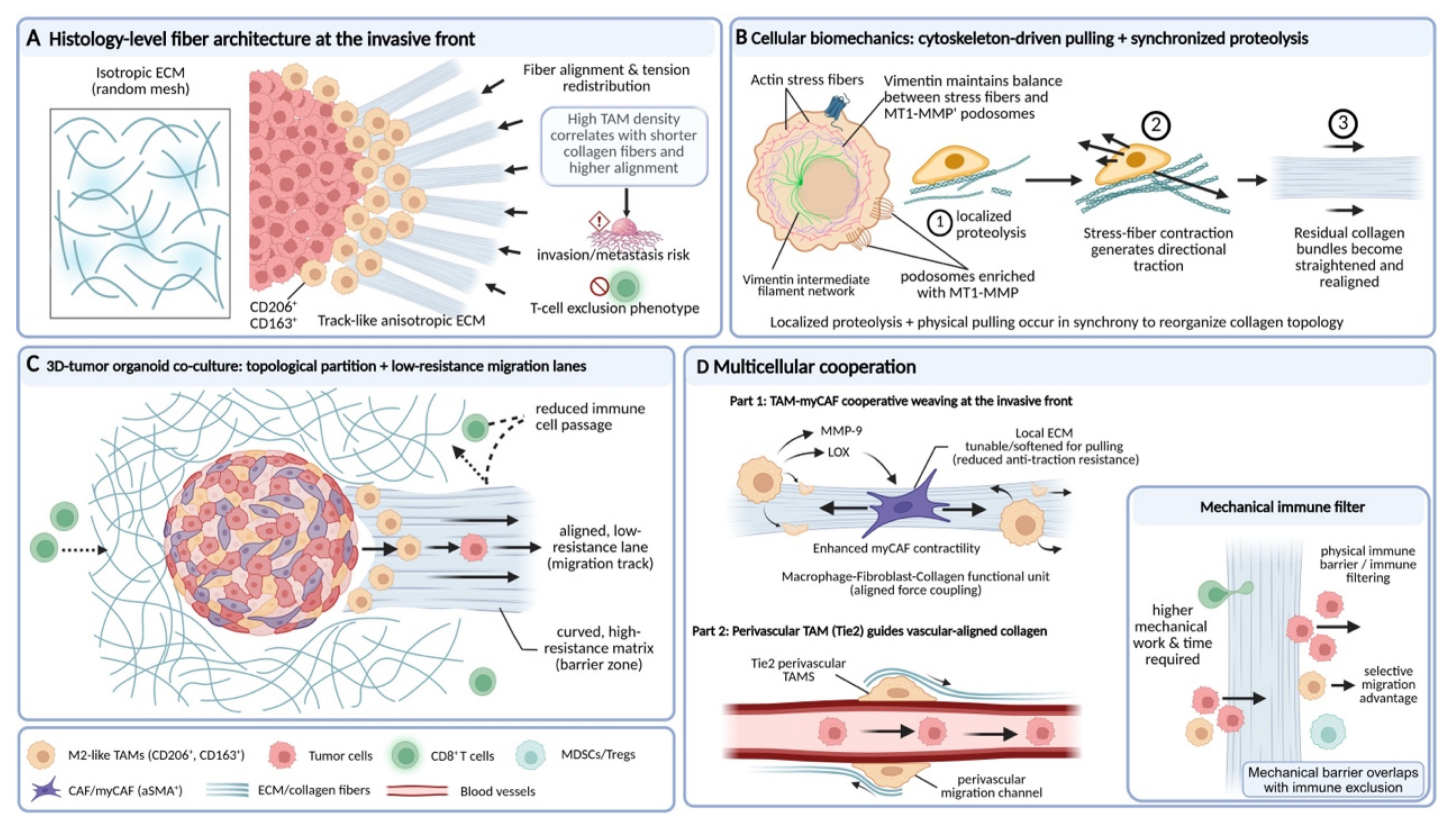
TAM-orchestrated collagen fiber alignment guides biomechanical signaling and establishes selective migration tracks at the invasive front. Schematic illustration of how TAMs reorganize collagen architecture to shape biomechanical signal guidance and immune exclusion. (A) At the tumor invasive front, high densities of CD206⁺/CD163⁺ TAMs associate with shortened, highly aligned collagen fibers, forming anisotropic, track-like ECM structures linked to increased invasion and T-cell exclusion. (B) At the cellular level, TAMs synchronize localized proteolysis with cytoskeleton-driven pulling, generating directional traction that straightens and realigns residual collagen bundles. (C) In 3D tumor organoid co-culture models, TAM-mediated fiber reorganization creates topologically partitioned matrices, opening low-resistance migration lanes for tumor cells while restricting immune cell passage. (D) Cooperative interactions between TAMs and myofibroblast-like CAFs, as well as perivascular TAMs, further reinforce aligned force coupling and vascular-guided collagen remodeling, collectively forming a mechanical immune filter that selectively favors tumor and myeloid cell migration over cytotoxic T-cell infiltration.

Importantly, the fibrous networks orchestrated predominantly by TAMs ultimately generate a highly selective physical microenvironment. This selectivity is reflected in the differential regulation of migratory capacities among distinct cell types. For instance, the reduction and heterogeneity of interfiber pore size markedly increase the mechanical work and time required for effector immune cells, such as CD8⁺ T cells, to traverse the matrix^59^. Their relatively rigid and larger cellular morphology results in inefficient migration within such dense and anisotropic structures. In contrast, tumor cells, TAMs themselves, and MDSCs, which possess greater deformability and matrix adaptability, are able to exploit—or even actively remodel—these channels more effectively^86^, ^87^. Consequently, the fiber rearrangement driven by TAMs effectively constructs a selective physical barrier that spatially integrates mechanical obstruction with immune exclusion, thereby coupling biomechanical constraints to immunosuppressive organization (**Fig. 2**).

### 2.3 Regulation of interstitial pressure

Abnormally elevated mechanical pressures within the TME constitute a critical physical barrier that restricts drug delivery and promotes immune evasion^2^. These pressures primarily comprise three interrelated forms: solid stress generated by tumor volumetric expansion and matrix contraction^88^, IFP arising from impaired vascular and lymphatic function^89^, and aberrant stress transmission and relaxation resulting from altered material properties of the ECM^90^. Accumulating evidence indicates that TAMs play indispensable and active roles in the generation, maintenance, and amplification of these distinct pressure components, rather than serving merely as passive responders to the mechanical milieu.

TAMs are central regulatory cells driving matrix fibrosis and stiffening, thereby generating and amplifying solid stress. M2-like TAMs secrete key mediators such as TGF-β and IL-10^11^, which not only directly stimulate collagen synthesis and the expression of cross-linking enzymes within TAMs themselves, but, more importantly, robustly activate CAFs, inducing their differentiation toward an α-smooth muscle actin (α-SMA)-expressing myofibroblastic phenotype^51^, ^91^. Activated CAFs produce large amounts of type I and type III collagen, hyaluronic acid, and other ECM components, while concomitantly upregulating cross-linking enzymes such as LOX, collectively resulting in marked increases in matrix stiffness and physical volume^6^, ^92^. Notably, ECM thickening and enhanced cross-linking substantially limit the compressibility of tumor tissue during growth. This structural rigidification leads to the accumulation of solid stress within the tumor core and perivascular regions, thereby compressing microvessels, reducing blood perfusion, and promoting the formation of hypoperfused and hypoxic areas^93^. These hypoxic regions, in turn, further reinforce M2-like TAM polarization and the expression of ECM biosynthetic programs through hypoxia-inducible factor-1α (HIF-1α)-dependent signaling, establishing a self-reinforcing positive feedback loop linking tissue stiffness, interstitial pressure, hypoxia, and TAM activation^12^, ^94^.

At the level of fluid dynamics, TAMs are also major drivers of elevated IFP within tumors, primarily through disruption of normal vascular and lymphatic function. On the one hand, TAMs—particularly perivascularly enriched Tie2⁺ subsets—are a major source of VEGF-A. The structurally disorganized and highly permeable neovasculature promoted by TAM-derived VEGF-A leads to continuous extravasation of protein-rich plasma components into the interstitial space^95^, ^96^. On the other hand, TAM-driven fibrosis and the associated solid stress directly compress and damage the already fragile intratumoral lymphatic vessels, severely impairing interstitial fluid drainage and clearance^11^, ^25^. In parallel, matrix densification and pore size reduction mediated cooperatively by TAMs and CAFs markedly decrease the mobility of water and solutes within the interstitium, increasing hydraulic resistance and fluid retention time, thereby sustaining elevated IFP^35^, ^97^, ^98^. Studies in animal models of pancreatic cancer and other solid tumors have demonstrated that increased local collagen content and structural complexity are positively correlated with total tissue pressure and are associated with vascular collapse and a pronounced reduction in chemotherapeutic drug uptake^99^. Conversely, localized administration of collagenase can acutely reduce tissue pressure and restore drug perfusion without altering overall tumor volume, directly demonstrating that collagen accumulation and cross-linking per se are key drivers of interstitial hypertension and heterogeneous drug distribution^100^-^102^.

**Figure 3:**
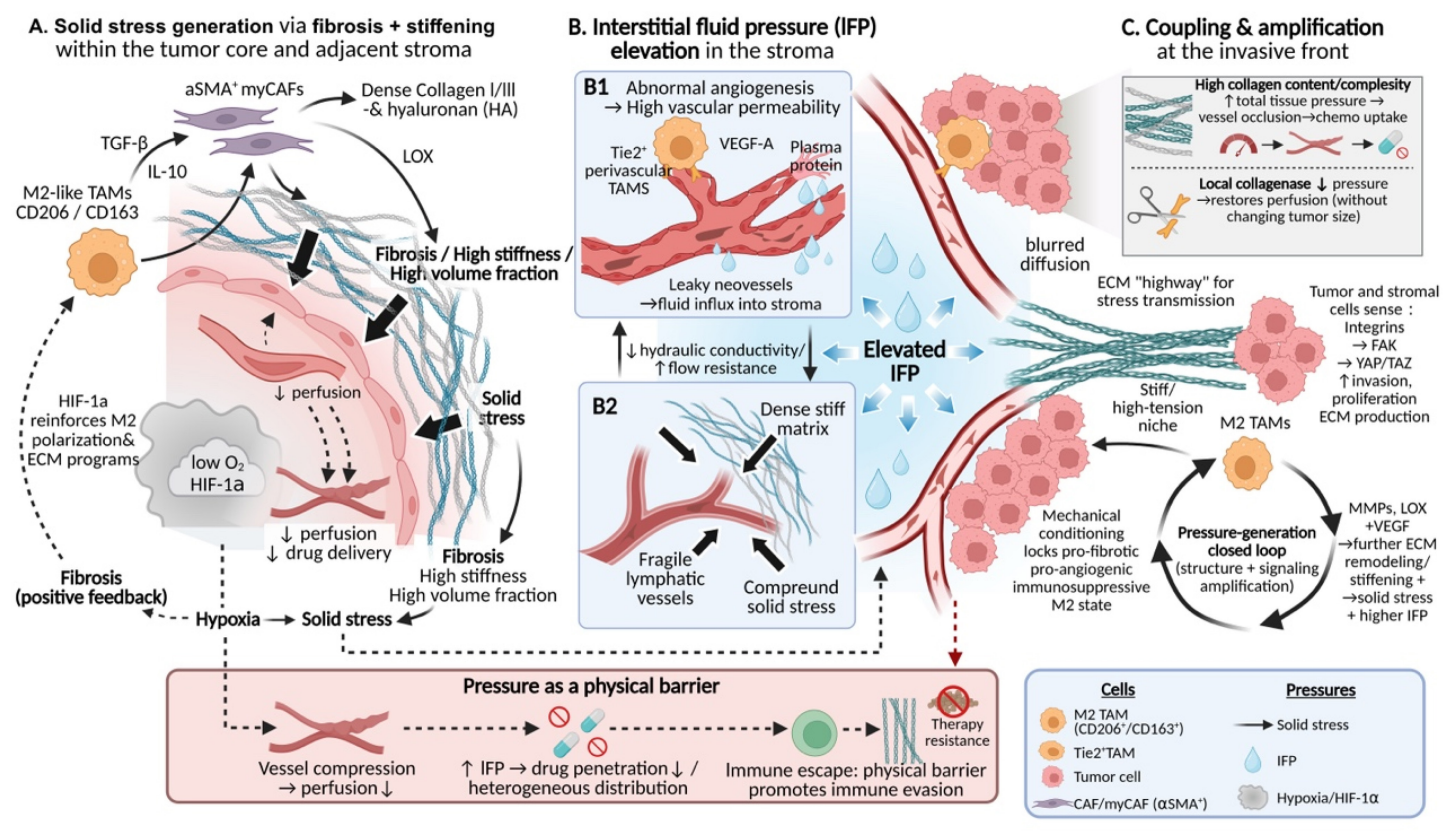
TAM-driven interstitial pressure as a physical barrier in tumors. Schematic illustration of how TAMs generate and amplify interstitial pressure within the TME. (A) M2-like TAMs promote fibrosis and matrix stiffening by activating αSMA⁺ myofibroblastic CAFs via TGF-β and IL-10, leading to collagen accumulation, solid stress generation, vascular compression, and hypoxia-driven positive feedback. (B) TAMs elevate IFP by inducing leaky angiogenesis through VEGF-A secretion and impairing lymphatic drainage, while dense ECM increases hydraulic resistance and fluid retention. (C) At the invasive front, stiff, collagen-rich matrices couple solid stress with fluid pressure and transmit mechanical signals sensed via integrin-FAK-YAP/TAZ pathways, reinforcing pro-fibrotic and immunosuppressive TAM states and establishing a self-amplifying pressure loop that restricts drug penetration and immune cell infiltration.

More importantly, the biological activities of TAMs tightly couple solid stress with fluid pressure and endow this system with potent signal amplification capacity. The ECM remodeled cooperatively by TAMs and CAFs serves not only as a source of mechanical pressure but also as an efficient conduit for the propagation of aberrant mechanical signals throughout tumor tissue. Dense and highly aligned collagen fiber networks enable more effective transmission of localized solid stress to distant cells, while elevated IFP influences the spatial distribution of chemical gradients by modulating convection and diffusion processes^97^, ^103^. Tumor cells and stromal cells sense these mechanical cues through mechanotransduction pathways involving integrins, FAK, and downstream effectors such as YAP/TAZ, which in turn induce enhanced invasive, proliferative, and ECM-synthetic programs^8^, ^104^. Notably, TAMs themselves constitute a core component of this mechanical feedback circuitry. High-stiffness and high-tension microenvironments actively promote the stabilization of TAMs toward pro-fibrotic, pro-angiogenic, and immunosuppressive M2-like phenotypes through the same mechanotransduction pathways^105^, ^106^. These TAMs subsequently secrete increased levels of matrix-modifying factors (e.g., MMPs and LOX) and pro-angiogenic mediators (e.g., VEGF), further driving matrix stiffening, contraction, and vascular leakage^107^, ^108^. Together, these processes culminate in a pressure-generating loop that is self-sustaining and self-amplifying at both structural and signaling levels (**Fig. 3**).

### 2.4 Mechanotransduction and signal amplification

Within the mechanically aberrant TME, TAMs do not merely passively respond to ECM stiffness and pressure. Instead, they translate local physical perturbations into systemic and sustained transcriptional and immunological reprogramming signals. This process of “amplification” enables TAMs to function as a central hub linking the physical microenvironment to biochemical responses and represents one of the core mechanisms underlying the maintenance of a highly fibrotic and immunosuppressive malignant steady state.

The initiation of this amplification process relies on diverse mechanosensors expressed on the surface of TAMs, including integrin clusters and mechanosensitive ion channels such as Piezo1 and TRPV4. Acting as highly sensitive antennas, these sensors continuously detect the physical properties of the microenvironment^109^. Upon signal input, mechanotransduction rapidly converges on key intracellular nodes, including FAK, proline-rich tyrosine kinase 2 (PYK2), and Rho GTPases^110^, ^111^, thereby driving the activation of downstream mechanotransduction pathways such as Hippo-YAP/TAZ signaling^104^. Notably, recent studies suggest that PYK2 not only occupies a central position in the transduction of integrin- and mechanics-derived signals, but also couples inflammatory and differentiation-related pathways, positioning it as a critical coordinator linking mechanical signal input to immune phenotypic polarization^112^, ^113^. Concurrently, increased stiffness and tensile forces suppress the Hippo kinase cascade, thereby promoting the nuclear translocation of YAP/TAZ^114^. Once translocated into the nucleus, YAP/TAZ drive the transcription of a series of target genes, including connective tissue growth factor (CTGF), cysteine-rich angiogenic inducer 61 (CYR61), TGF-β, and LOX^115^, ^116^, effectively converting the physical stiffness of the matrix into explicit biochemical instructions that promote fibrosis and angiogenesis^117^.

More importantly, mechanically induced phenotypic changes in TAMs are not transient events, but are accompanied by profound epigenetic remodeling. Accumulating evidence indicates that YAP/TAZ and their co-activators can function as scaffolds to recruit histone acetyltransferases and methyltransferase complexes, such as p300, to the promoters and enhancers of mechanosensitive genes, catalyzing active chromatin modifications including H3K27ac and H3K4me1, thereby maintaining an open chromatin state at these loci^118^-^120^. This process, initiated by mechanical cues and ultimately consolidated into a defined epigenetic landscape, provides a stable molecular basis for the sustained pro-tumorigenic functions of TAMs in a dynamically changing microenvironment and partially explains the persistence and self-maintaining nature of fibrotic tumor niches.

**Figure 4:**
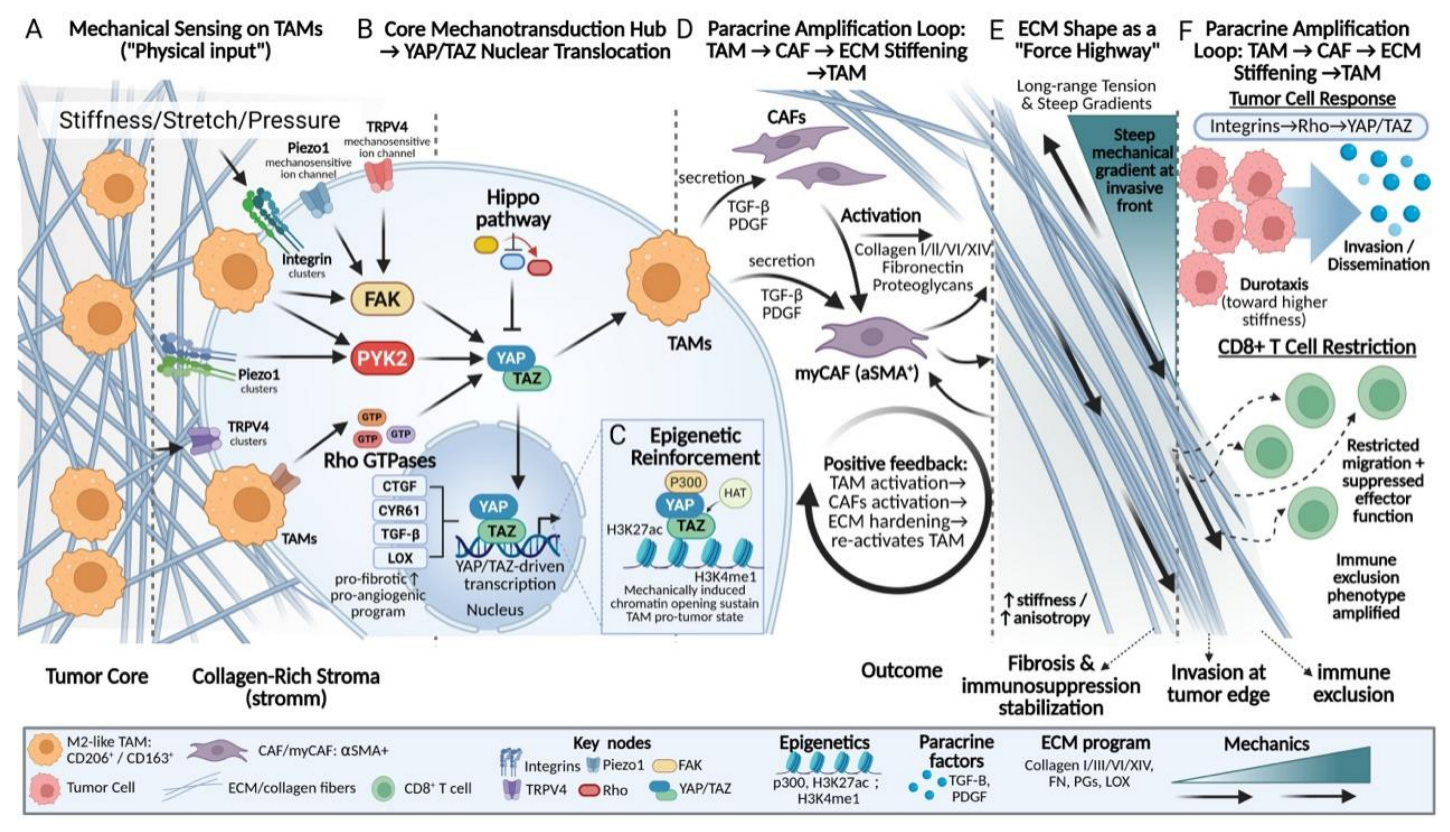
Mechanotransduction and signal amplification in TAMs within stiff tumor matrices. Schematic overview of how TAMs convert mechanical cues into sustained pro-fibrotic and immunosuppressive programs. (A-B) Increased matrix stiffness, stretch, and pressure are sensed by integrins and mechanosensitive ion channels (Piezo1, TRPV4), converging on FAK-PYK2-Rho signaling to activate YAP/TAZ nuclear translocation. (C) YAP/TAZ-driven transcription is stabilized by epigenetic reinforcement, including p300-mediated H3K27ac and H3K4me1, sustaining TAM pro-tumor states. (D-F) Paracrine activation of CAFs by TAM-derived TGF-β and PDGF promotes ECM deposition, alignment, and stiffening, forming a positive feedback loop. Aligned collagen fibers act as “force highways,” amplifying mechanical gradients that drive tumor cell durotaxis while restricting CD8⁺ T-cell migration, thereby reinforcing invasion and immune exclusion.

Following the completion of their own mechanical signal transduction and phenotypic reprogramming, TAMs propagate the amplified mechanical signals throughout the microenvironment in a paracrine manner. The secretion of key cytokines, such as TGF-β and PDGF, constitutes a central signal for the activation and maintenance of CAFs^121^. Activated CAFs, in turn, synthesize and deposit large amounts of ECM components, including type I and type III collagen, thereby markedly increasing ECM stiffness and mechanical anisotropy^37^. The resulting increase in matrix stiffness subsequently feeds back through mechanosensors such as integrins to further reinforce the pro-fibrotic phenotype of TAMs, forming a self-sustaining malignant feedback loop^38^. Notably, TAM-mediated shaping of tumor mechanical properties is not solely dependent on cytokine secretion, but is also achieved through direct physical remodeling of ECM spatial architecture. The linearly aligned and densely packed collagen fiber bundles formed with TAM participation exhibit higher elastic moduli and are more efficient at transmitting contractility-generated mechanical tension^122^. Such specialized ECM structures enable long-range transmission and focalization of local stress along fiber axes, thereby generating steep mechanical gradients at critical regions such as the tumor-stroma interface and the invasive front^62^. Tumor cells can sense these mechanical gradients through their integrin-Rho-YAP/TAZ axis and subsequently undergo directed durotactic migration, invading surrounding tissues along high-stiffness fiber tracks^35^, ^123^, ^124^. In contrast, effector immune cells, such as CD8⁺ T cells, exhibit severely restricted migration within these high-tension and structurally heterogeneous fibrous networks, accompanied by impaired activation and effector functions^11^, ^59^. Through these mechanisms, TAMs synergistically exacerbate tumor immune exclusion at both physical and immunological levels (**Fig. 4**).

## 3. Reciprocal regulation of TAMs by the biomechanical microenvironment

### 3.1 Mechanical sensing and initial signal transduction in TAMs

During tumor progression, ECM stiffening is not merely a passive background alteration but functions as a potent physical cue that is actively sensed by TAMs and their precursor cells. The decoding of mechanical signals is initiated at the plasma membrane, where collagen network reorganization induced by matrix stiffening increases the tensile load borne by integrin clusters and mechanosensitive ion channels such as Piezo1.

Studies have demonstrated that, in hydrogels mimicking the high stiffness of pancreatic cancer and other solid tumors, monocytes rapidly respond to matrix stiffness of approximately 30 kPa through Piezo1-mediated Ca^2+^ influx^125^, ^126^. This influx activates downstream signaling pathways, including calmodulin-calpain, mitogen-activated protein kinase (MAPK), and HIF-1α^127^, ^128^. These early events fundamentally reshape the inflammatory cytokine secretion profile and metabolic gene expression programs of macrophages, thereby influencing their differentiation trajectory^129^. In parallel, integrin engagement with ECM ligands induces focal adhesion assembly, recruiting proteins such as talin, vinculin, and FAK to form a mechanically coupled signal transduction platform linked to the cytoskeleton^130^.

These membrane-proximal events are further integrated within the cytoplasm. Piezo1-Ca²⁺ signaling cooperates with integrin β1-mediated actin traction to activate the immunomechanical checkpoint PYK2^131^. Concurrently, integrin cluster-activated FAK further engages Rho GTPase signaling, which suppresses the Hippo pathway and promotes dephosphorylation and nuclear translocation of its downstream effectors YAP/TAZ^132^, ^133^.

### 3.2 Mechanotransductive phenotypic remodeling and stabilization of TAM programs

Following the initial sensing and transduction of mechanical cues, these signals are progressively integrated into gene regulatory programs that drive stable phenotypic remodeling of TAMs.

In pancreatic cancer models, PYK2 not only integrates upstream mechanical inputs derived from Piezo1 and integrins, but also drives F-actin polymerization and undergoes nuclear translocation^134^. This directly regulates the promoters of mechanosensitive and differentiation-associated genes, such as ACTR3 and RELA, thereby determining both the efficiency of monocyte-to-TAM differentiation and the direction of macrophage polarization. Genetic or functional ablation of PYK2 markedly impairs the generation of monocyte-derived TAMs and their immunosuppressive activity, while enhancing the therapeutic efficacy of programmed cell death protein 1 (PD-1) blockade, underscoring its central role in mechanically driven macrophage polarization^113^.

**Figure 5:**
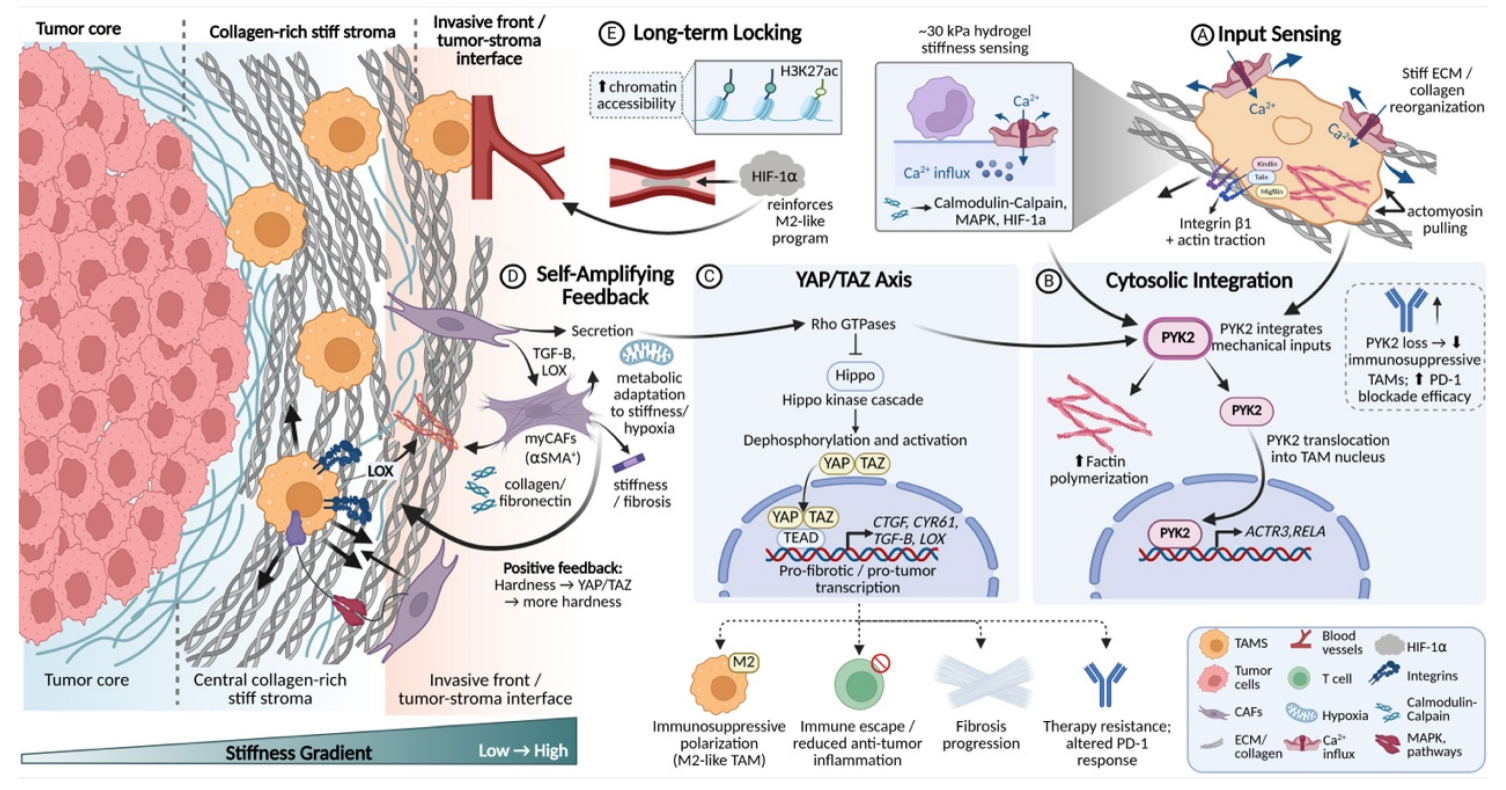
Mechanotransductive programming of TAM phenotypes along tumor stiffness gradients. Schematic illustration of how ECM stiffening instructs TAMs phenotypic programming. (A) At collagen-rich stiff stroma and the invasive front, reorganized ECM increases tensile load on integrins and activates mechanosensitive ion channels, including Piezo1, inducing Ca²⁺ influx. (B) Mechanical inputs are integrated through integrin β1-actomyosin traction and the mechanosensitive checkpoint PYK2, promoting cytoskeletal remodeling and nuclear signaling. (C) PYK2- and FAK-dependent activation of Rho GTPases suppresses Hippo signaling and drives YAP/TAZ nuclear translocation, initiating pro-fibrotic and immunosuppressive transcriptional programs. (D) TAM-derived TGF-β and LOX activate myCAFs, enhancing ECM deposition and stiffening. (E) YAP/TAZ-associated epigenetic remodeling locks TAMs into stable M2-like states, establishing a self-amplifying stiffness-mechanotransduction feedback loop.

Nuclear translocation of YAP/TAZ represents a pivotal step in converting mechanical signals into sustained transcriptional programs. Across multiple tumor models, including breast and colorectal cancer, macrophages cultured on rigid substrates exhibit significantly increased nuclear localization of YAP/TAZ^135^, ^136^, which correlates with enrichment of M2-like TAMs and poor patient prognosis^137^. Once in the nucleus, YAP/TAZ function as transcriptional co-activators in cooperation with TEAD family proteins^119^, robustly inducing the expression of genes such as CTGF, CYR61, TGF-β, and LOX^138^. The resulting production of these factors further exacerbates ECM cross-linking and fibrosis^139^, thereby establishing a self-reinforcing loop in which matrix stiffness initiates YAP/TAZ signaling, which is subsequently amplified to drive further matrix stiffening and progressive magnification of the original mechanical input^140^, ^141^. More profoundly, the effects of this pathway are inscribed into long-term TAM functional memory through epigenetic remodeling and metabolic reprogramming. Specifically, YAP/TAZ regulate chromatin accessibility, enabling sustained high-level expression of mechanosensitive genes even after mechanical stimulation diminishes^142^, ^143^. In parallel, metabolic reprogramming jointly mediated by Piezo1-dependent Ca^2+^ signaling and YAP/TAZ endows TAMs with metabolic traits adapted to high-stiffness and hypoxic microenvironments^126^, ^144^. Thus, through the integrin/Piezo1-PYK2-YAP/TAZ axis, the tumor matrix systematically decodes physical stiffness into biological instructions that drive and stabilize specific TAM polarization states (**Fig. 5**).

### 3.3 Physical barrier-driven metabolic and functional output

Following mechanically driven phenotypic remodeling, TAMs translate these internal transcriptional changes into profound metabolic and functional outputs. In densely fibrotic tumors, physical barriers formed by aberrantly deposited collagen and elevated IFP exert effects that extend beyond simply altering cellular mechanosensing. More importantly, these barriers reshape the spatiotemporal distribution of nutrients, metabolic substrates, and signaling molecules within the TME, thereby driving characteristic metabolic reprogramming in TAMs.

TAMs themselves are active architects of the fibrotic barrier, a process that is tightly coupled to their own metabolic remodeling. Recent studies have shown that, under conditions of matrix stiffening and TGF-β signaling, TAMs can initiate intrinsic collagen biosynthetic programs, upregulating the expression of type I collagen and key enzymes such as prolyl hydroxylases^145^, ^146^. Collagen biosynthesis is highly dependent on arginine metabolism^147^, ^148^, leading TAMs to consume large amounts of arginine from the microenvironment and convert it into proline and ornithine. As a direct consequence, extracellular arginine concentrations are markedly reduced in TAM-enriched regions, whereas levels of proline and ornithine are increased^148^, ^149^. Arginine depletion directly suppresses the proliferation and function of CD8⁺ T cells, as T-cell activation and expansion are strictly dependent on this amino acid^149^, ^150^. Meanwhile, accumulated ornithine can be further converted into polyamines, which not only promote tumor cell proliferation and angiogenesis but also serve as important facilitators of collagen cross-linking, thereby synergistically exacerbating matrix stiffening at both metabolic and structural levels^147^, ^151^.

Diffusion restriction and impaired perfusion imposed by physical barriers place TAMs under a distinctive and sustained metabolic stress environment, characterized by chronic hypoxia, limited glucose availability, and concomitant enrichment of metabolic by-products such as lactate and fatty acids^152^, ^153^. To adapt to this environment, TAMs undergo a fundamental metabolic shift, transitioning from the glycolysis-dependent pro-inflammatory phenotype toward a greater reliance on oxidative phosphorylation and fatty acid oxidation^154^-^156^. For example, large amounts of lactate produced by tumor cells and stromal cells can be taken up by TAMs and utilized to fuel oxidative phosphorylation^157^. At the molecular level, this metabolic transition is associated with activation of signaling pathways such as STAT3 and peroxisome proliferator-activated receptor δ (PPARδ), which together stabilize the immunosuppressive gene expression program of TAMs, including upregulation of programmed death-ligand 1 (PD-L1) and high expression of ARG1 and IL-10^157^, ^158^. Metabolically reprogrammed TAMs further promote aberrant angiogenesis and matrix deposition through the secretion of factors such as VEGF and TGF-β, leading to poorer tissue perfusion and more restricted molecular diffusion, thereby reinforcing the adverse microenvironment that initially drove their metabolic adaptation^159^, ^160^.

**Figure 6:**
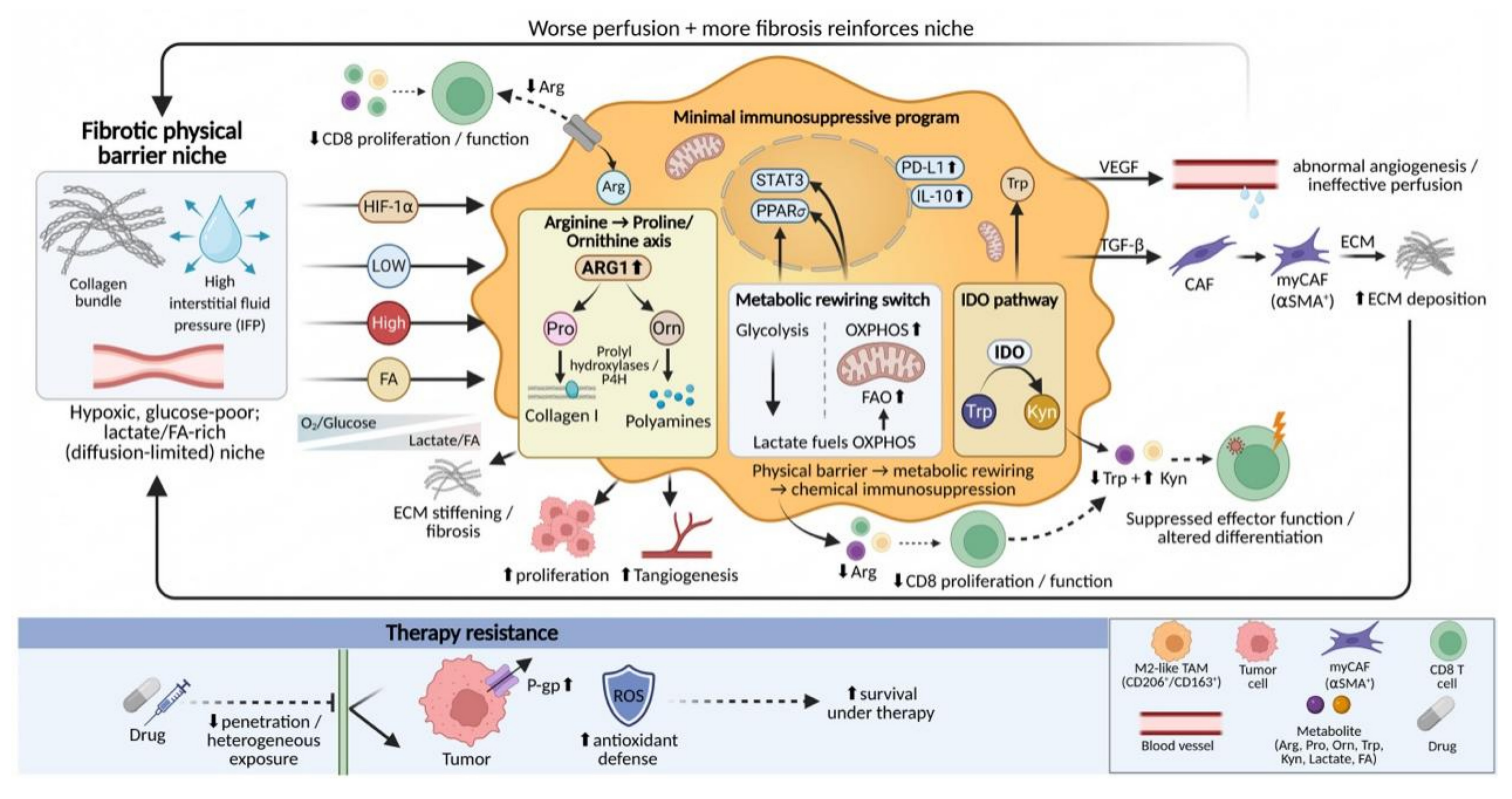
Fibrotic physical barriers mechanotransductively program TAM metabolism to enforce immune exclusion and therapy resistance. Dense collagen bundles and elevated IFP restrict diffusion and impair perfusion, creating a hypoxic, glucose-poor but lactate/FA-rich niche. TAMs adapt by shifting from glycolysis toward OXPHOS/FAO (lactate-fueled) and activating STAT3/PPARδ, reinforcing an immunosuppressive program (PD-L1, IL-10, ARG1). In stiff, TGF-β-rich regions, TAMs also engage an arginine→proline/ornithine axis to support collagen maturation (prolyl hydroxylases) and generate ornithine-derived polyamines that promote tumor growth, angiogenesis, and collagen cross-linking, further stiffening ECM. Metabolic outputs superimpose a chemical barrier: ARG1-driven arginine depletion and IDO-mediated Trp to Kyn suppress CD8⁺ T-cell function. TAM-derived VEGF/TGF-β sustains abnormal angiogenesis, myCAF activation, ECM deposition, limited drug penetration, and TAM-assisted survival under therapy.

Beyond passive adaptation, TAMs actively convert physical obstruction into functional immune exclusion through their metabolic outputs. Within dense matrix regions, ARG1-high TAMs exacerbate local arginine depletion^149^. In addition, TAMs can deplete tryptophan through the indoleamine 2,3-dioxygenase (IDO) pathway, generating immunosuppressive metabolites such as kynurenine^161^, ^162^. These metabolic activities superimpose a chemical barrier onto the physical barrier, systemically suppressing effector immune cell function and influencing their differentiation fate^163^. This metabolic remodeling process also represents one of the central mechanisms underlying therapeutic resistance. Dense stroma impedes the uniform diffusion of therapeutic agents, while M2-like TAMs localized within these regions promote tumor cell survival under drug exposure by upregulating drug efflux pumps, such as P-glycoprotein, and enhancing antioxidant metabolic capacity^161^, ^164^ (**Fig. 6**).

### 3.4 Mechanically guided spatial distribution and accumulation

Ultimately, the cascade of mechanical sensing and functional remodeling culminates in the precise spatial distribution of TAMs. Within tumors, heterogeneity in ECM stiffness, the preferential alignment of collagen fibers, and local differences in tissue pressure collectively generate a complex mechanical microenvironment. By regulating TAM adhesive properties, migratory behavior, and ultimate positioning, this mechanical landscape drives TAM enrichment in specific functional niches—such as the invasive front, perivascular regions, or highly fibrotic tumor cores—thereby establishing a suppressive microenvironmental architecture in which spatial distribution is tightly coupled to function.

Highly aligned collagen fiber bundles within the tumor stroma provide structural physical tracks that support directed TAM migration^165^, ^166^. This process depends on the active sensing and response of cells to the physical properties of the matrix. Integrin-mediated cell-matrix adhesion transduces information regarding fiber orientation and tension into intracellular signals that govern actin cytoskeletal reorganization through focal adhesion complexes^167^. When a precise balance is achieved between local adhesion strength and actomyosin contractility-generated traction forces, TAMs preferentially undergo persistent, directional migration along the longitudinal axis of collagen fibers rather than random movement^168^, ^169^. Experimental evidence demonstrates that, in regions with matrix stiffness of approximately 8-12 kPa and highly ordered fiber alignment, both the migration speed and directionality of macrophages are significantly enhanced^170^-^172^. This enables TAMs to migrate systematically along fibrotic tracks pre-remodeled by tumor cells or CAFs, ultimately accumulating at mechanically active tumor-stroma interfaces.

Notably, the molecular mechanisms governing TAM migration and retention frequently integrate classical immune signaling pathways with mechanotransduction circuits, thereby employing distinct modalities to coordinate the sequential processes of directional navigation and site-specific anchoring. Recent studies have revealed a direct role for innate immune signaling in regulating macrophage mechanical behavior. Specifically, mechanical stress within the TME can activate the cyclic GMP-AMP synthase (cGAS)-stimulator of interferon genes (STING)-TANK-binding kinase 1 (TBK1) signaling axis in TAMs^173^. Activated TBK1 directly phosphorylates the focal adhesion protein zyxin, thereby strengthening its association with the actin cytoskeleton and stabilizing focal adhesion structures. This signaling cascade ultimately suppresses macrophage motility and promotes their long-term retention within tumor tissue. Genetic or pharmacological disruption of this pathway effectively reduces intratumoral TAM accumulation and enhances the efficacy of immune checkpoint blockade therapy^174^. These findings illustrate that immune signaling pathways can directly determine TAM spatial distribution by modulating the physical properties of the cytoskeleton. Similarly, the initial recruitment of monocytes is regulated by coupled mechanical and chemical cues^113^. For example, the matricellular protein spondin-2 (SPON2), secreted by tumor cells, can bind integrin β1 on the surface of monocytes, activating downstream PYK2 signaling and markedly enhancing cellular adhesion and transendothelial migration^175^-^177^, thereby driving monocyte infiltration into tumor tissue^178^.

Interestingly, the integrin-PYK2 axis and the cGAS-STING-TBK1 axis converge on Zyxin, a key regulator of actin dynamics and focal adhesion turnover, yet impose markedly divergent functional outputs. Activation of the integrin-PYK2 pathway promotes the recruitment of Zyxin to nascent focal adhesions at the leading edge, thereby accelerating adhesion turnover and supporting efficient, directional migration along ECM fibers^179^, ^180^. In contrast, TBK1-mediated phosphorylation of Zyxin at Ser143 enhances its localization to mature focal adhesions, stabilizing these structures and attenuating actin cytoskeletal dynamics, ultimately constraining TAMs in a sessile, tissue-resident state^181^. Thus, rather than operating in a purely competitive or cooperative manner, these pathways are functionally integrated: PYK2-driven signaling directs TAMs toward specific mechanical niches, whereas TBK1-Zyxin signaling stabilizes their retention at these sites, together ensuring precise spatial positioning of TAMs within the heterogeneous mechanical landscape of the tumor microenvironment.

**Figure 7:**
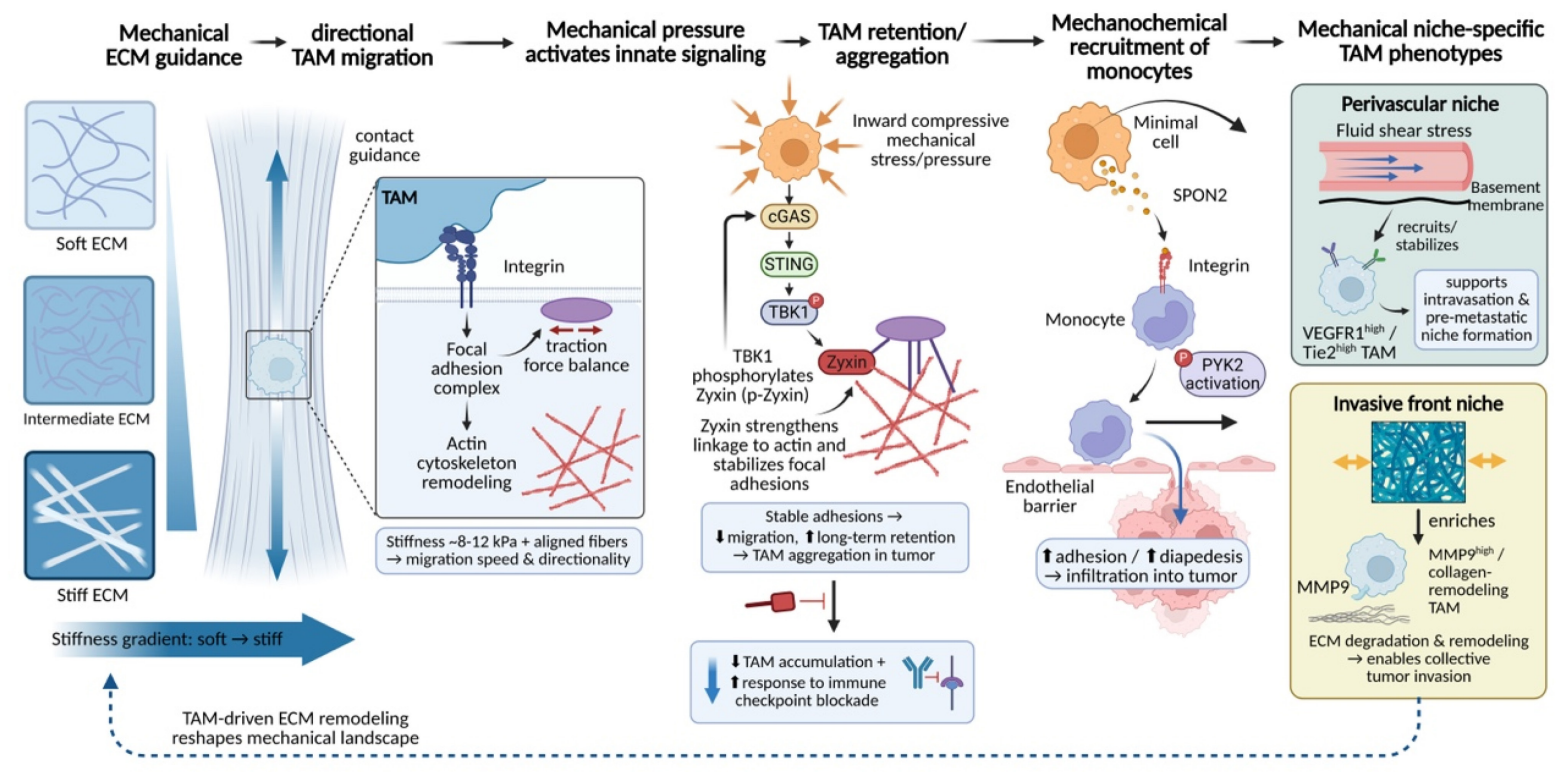
Mechanically driven TAM migration and spatial accumulation establish niche-specific TAM programs. ECM stiffness gradients and collagen fiber alignment provide mechanical guidance cues that promote persistent, directional TAM migration via integrin-focal adhesion signaling and actin remodeling, with maximal speed/directionality in ~8-12 kPa, highly ordered matrices. Local compressive stress activates the innate cGAS-STING-TBK1 axis; TBK1 phosphorylates zyxin to strengthen actin coupling and stabilize adhesions, suppressing motility and promoting long-term TAM retention/aggregation—an effect whose disruption reduces TAM accumulation and improves checkpoint blockade responses. Mechanical cues also cooperate with chemotactic signals to recruit monocytes: tumor-derived SPON2 engages integrin β1, activates PYK2, and enhances adhesion, diapedesis, and infiltration. Distinct mechanics then select functional TAM states, including perivascular VEGFR1-high/Tie2-high TAMs (shear/basement membrane; intravasation, pre-metastatic niche) and invasive-front MMP9-high collagen-remodeling TAMs enabling collective invasion.

The ultimate outcome of such mechanically guided processes is the establishment of finely tuned functional spatial organization of TAMs within tumors. TAM subpopulations residing in microenvironments with distinct mechanical characteristics exhibit markedly different phenotypes and functions. In perivascular regions, unique combinations of fluid shear stress and basement membrane components preferentially recruit and stabilize TAMs with high expression of receptors such as VEGFR1 and Tie2^25^, ^182^, ^183^. These cells play critical roles in facilitating tumor cell intravasation and the formation of pre-metastatic niches^183^, ^184^. In contrast, at the tumor invasive front, high tensile forces and densely cross-linked ECM selectively enrich TAM subsets characterized by high expression of MMP9 and collagen remodeling-associated genes^34^, ^185^, with functions centered on ECM degradation and restructuring to enable collective tumor cell migration^186^-^188^. Thus, local mechanical properties act as a selective pressure that shapes both the spatial and functional heterogeneity of the TAM population. Importantly, TAMs do not merely respond passively to pre-existing mechanical environments; as described above, they actively participate in the dynamic construction of the tumor mechanical landscape through continuous ECM remodeling activities (**Fig. 7**).

### 3.5 Self-Reinforcing Malignant Loop of Bidirectional Regulation

The bidirectional interplay between TAMs and the tumor mechanical microenvironment, as delineated in Sections 2 and 3, is not merely a sequential coupling of independent processes, but rather constitutes a self-reinforcing malignant system. In this system, TAM-driven remodeling of the ECM and mechanically induced TAM reprogramming are intrinsically interdependent, forming a closed-loop circuit that progressively stabilizes both stromal fibrosis and immunosuppression.

At the structural level, TAMs, in concert with CAFs, continuously reshape the ECM through coordinated deposition, crosslinking, degradation, and fiber reorganization, leading to increased matrix stiffness, elevated interstitial pressure, and the establishment of highly anisotropic collagen architectures. These alterations, in turn, redefine the mechanical landscape of the tumor microenvironment. At the signaling level, such persistent mechanical cues are sensed and transduced by TAMs through integrin-dependent adhesions and mechanosensitive pathways, thereby reinforcing pro-fibrotic and immunosuppressive transcriptional programs. At the functional level, the resulting TAM populations further amplify matrix remodeling, metabolic reprogramming, and immune exclusion, collectively consolidating a tumor-permissive niche.

Importantly, this feedback loop is not only molecularly self-sustaining but also spatially and temporally propagative. Mechanically defined niches characterized by high stiffness and dense fibrosis preferentially recruit and retain TAMs, while simultaneously imposing metabolic and physical constraints that suppress effector immune cell infiltration. As a consequence, newly recruited monocytes entering these regions are rapidly conditioned by the pre-established mechanical context, thereby perpetuating the expansion of pro-tumorigenic TAM populations.

Taken together, this self-reinforcing loop provides a unifying framework to understand how mechanical and immune dysregulation co-evolve during tumor progression. It also implies that disrupting this bidirectional coupling—rather than targeting individual components in isolation—may represent a critical prerequisite for effectively overcoming mechano-immunological resistance.

## 4. Therapeutic strategies

### 4.1 Targeting TAMs to mechanically soften tumors

Directly interfering with the pro-fibrotic activities of TAMs through strategies such as depletion, reprogramming, functional suppression, or signaling blockade can modulate tumor matrix stiffening at its cellular source. By alleviating aberrant stromal hardening, these approaches create a more permissive physical microenvironment for improved drug delivery and immune cell infiltration.

#### 4.1.1 TAMs depletion and inhibition of monocyte recruitment

Given that aberrant physical cues and chemotactic axes (e.g., CCL2-CCR2) continuously drive monocyte recruitment to fuel early stromal stiffening, depleting established TAM populations and blocking their influx represent foundational therapeutic strategies for alleviating tumor immunosuppression and matrix densification. The core of this approach lies in blocking key signaling axes, such as CCL2-CCR2 and colony-stimulating factor 1 (CSF-1)/CSF-1 receptor (CSF-1R), thereby directly reducing the abundance and supply of immunosuppressive TAMs and weakening their support for tumor growth, fibrosis, and tissue mechanical stiffening at the source.

With respect to suppressing monocyte recruitment to tumor sites, targeting the CCL2-CCR2 axis is among the most extensively investigated strategies^15^, ^189^. In stroma-rich tumors such as pancreatic ductal adenocarcinoma (PDAC), CCL2 is highly expressed by both tumor cells and stromal components. Through engagement of its receptor CCR2, CCL2 continuously recruits CCR2^+^ monocytes to the tumor, where they subsequently differentiate into TAMs^190^, ^191^. Preclinical studies have demonstrated that treatment with the CCR2 small-molecule inhibitor PF-04136309^192^ or the anti-CCL2 monoclonal antibody Carlumab^193^ significantly reduces intratumoral TAMs and MDSCs, while enhancing the efficacy of chemotherapy or immunotherapy. Notably, the redundancy of the chemokine network suggests that simultaneous targeting of multiple signaling axes may yield greater therapeutic benefit^194^. For example, in hepatocellular carcinoma models, blockade of the CCL5/CCR5 axis not only suppresses the recruitment and function of polymorphonuclear MDSCs, but also indirectly modulates the immunosuppressive TME^195^. Although early clinical trials of CCL2-CCR2 inhibitors failed to meet expectations due to limited efficacy or toxicity, these outcomes underscore the presence of complex compensatory mechanisms within the TME and have prompted the development of next-generation agents and combination strategies.

In contrast to inhibiting monocyte recruitment, direct elimination of established TAM populations is primarily achieved by targeting the CSF-1/CSF-1R signaling pathway, which is central to macrophage survival, proliferation, and differentiation^15^, ^196^ Studies have shown that small-molecule CSF-1R inhibitors, such as PLX3397 and BLZ945, markedly reduce the density of CD11b⁺F4/80⁺ TAMs across multiple murine solid tumor models, accompanied by enhanced CD8⁺ T-cell infiltration and reduced tumor burden^197^-^199^. Similarly, in head and neck squamous cell carcinoma models, BLZ945 and PLX3397 not only induce TAM apoptosis but also downregulate CD206 and IL-10 expression, thereby alleviating immunosuppression and synergizing with cisplatin to suppress tumor growth^200^, ^201^. In sonic hedgehog (SHH)-subtype medulloblastoma and sarcoma models, treatment with PLX5622 or PLX3397 likewise reduces TAM abundance and prolongs survival, indicating broad therapeutic potential of CSF-1R blockade in tumors highly dependent on TAMs^197^, ^202^. However, reduction in TAM numbers alone does not always translate into durable antitumor efficacy, in part because residual TAMs may undergo compensatory adaptation or promote the recruitment of alternative immunosuppressive cell populations^203^, ^204^. Accordingly, next-generation strategies aim to achieve more sustained and comprehensive remodeling of the TAM ecosystem. For instance, a recently reported covalent CSF-1R inhibitor, FF-10101, achieves prolonged and profound suppression of CSF-1R signaling through irreversible target engagement. In animal models, FF-10101 not only markedly reduces immunosuppressive TAMs but also expands TAM subsets with antitumor potential, while significantly enhancing antigen-specific CD8⁺ T-cell responses. Importantly, combination treatment with anti-PD-1 antibodies demonstrate pronounced synergistic antitumor effects^205^. Similarly, the next-generation CSF-1R inhibitor PXB17 exhibits superior pharmacokinetic properties and efficacy compared with PLX3397 in colorectal cancer models, significantly reducing M2-like TAMs and promoting CD8^+^ T-cell infiltration, thereby enhancing and stabilizing the therapeutic effects of PD-1 blockade^197^. It should be noted, however, that CSF-1R inhibitors are not universally effective as monotherapies across all tumor types and may exert adverse effects on dendritic cells and macrophages in normal tissues^197^, ^206^, ^207^. These findings indicate that TAM depletion or recruitment-inhibition strategies are more suitably applied in combination with ICIs or chemotherapy to effectively remodel the tumor immune microenvironment.

Beyond these canonical pathways, certain chemotactic signaling circuits with greater spatial selectivity offer additional opportunities for precision intervention. For example, in triple-negative breast cancer, the interleukin-17A (IL-17A)-osteopontin (OPN)-LYVE-1 axis has been shown to coordinately regulate the accumulation of TAMs from distinct origins. Specifically, IL-17A-induced OPN recruits peripheral monocytes through integrins such as αvβ3, promoting their differentiation into pro-fibrotic TAMs, while simultaneously stimulating the proliferation of tissue-resident macrophages^208^. Targeting such pathways may enable selective suppression of fibrosis-driving TAM subsets without complete macrophage depletion (**Table 1**).

#### 4.1.2 TAMs reprogramming

Because elevated matrix stiffness actively engages mechanotransduction networks to lock macrophages into a pro-fibrotic M2-like state, therapeutic reprogramming aims to counteract this mechanically driven polarization by redirecting TAMs toward an M1-like state with antitumor functions. Rather than eliminating TAMs, this strategy fundamentally alters their functional identity, thereby dismantling their support for the tumor mechanical microenvironment at its core.

Agonist-based approaches initiate reprogramming by activating innate immune signaling pathways. Among these, agonistic CD40 antibodies represent one of the most advanced strategies. CD40, a member of the tumor necrosis factor receptor superfamily, effectively drives macrophage polarization toward an M1-like phenotype upon activation and upregulates molecules involved in antigen presentation^209^-^211^. In fibrotic tumor models such as PDAC, the CD40 agonist APX005M not only induces phenotypic conversion of TAMs but also triggers pronounced stromal remodeling^212^. This includes reduced collagen density, improved vascular perfusion, and concomitant deep infiltration of CD8^+^ T cells^213^. Clinical studies further demonstrate that CD40 agonists combined with chemotherapy induce tumor stromal alterations and elicit T-cell responses in patients with PDAC^214^. Toll-like receptor (TLR) agonists constitute another important class of reprogramming agents. Encapsulation of the TLR7/8 agonist resiquimod (R848) into CD206-targeting nanoparticles enables selective activation of TLR signaling within TAMs. In breast cancer models, this targeted delivery system successfully reprograms TAMs toward an M1 phenotype and significantly suppresses pulmonary metastasis. Such targeted delivery strategies enhance therapeutic efficacy while minimizing systemic toxicity associated with widespread immune activation^215^. Notably, TAM reprogramming exhibits pronounced temporal dependence. M1-like states induced by CD40 or TLR agonists tend to revert to mixed or suppressive phenotypes in the absence of sustained signaling^216^, ^217^. This limitation indicates that molecular agonists alone are insufficient to maintain long-term functional conversion of TAMs and provides a rationale for integrating reprogramming strategies with delivery systems or biomaterial-based platforms.

Adoptive cell therapies, particularly chimeric antigen receptor macrophage (CAR-M) technologies, offer an engineered solution for TAM reprogramming. Through genetic modification, macrophages are endowed with CAR constructs that enable specific recognition of tumor antigens. Beyond conferring targeted cytotoxicity, CAR signaling itself drives macrophages toward a pro-inflammatory, antigen-presenting state^218^. Preclinical studies of human anti-HER2 CAR-M cells (CT-0508) demonstrate that infused CAR-Ms efficiently infiltrate tumors and remodel the immunosuppressive TME into a pro-inflammatory state, as evidenced by increased expression of M1 markers and pro-inflammatory cytokines^219^. Notably, CAR-Ms can also secrete MMPs, directly degrading dense ECM and thereby physically reducing tumor stiffness^49^, ^220^. Based on these findings, CT-0508 has advanced into a phase I clinical trial (NCT04660929), with preliminary data indicating favorable safety profiles and early signs of TME remodeling^221^. In addition, more advanced cellular therapies are under exploration, including macrophages engineered to release encapsulated reprogramming factors only in response to specific tumor microenvironmental cues, such as low pH or elevated reactive oxygen species, enabling safer and more precise in situ phenotypic conversion^222^.

Metabolic and epigenetic interventions are critical for stabilizing reprogrammed phenotypes and preventing reversion to M2-like states. Metabolites within the TME are key drivers maintaining the M2 phenotype of TAMs^223^, and targeting these metabolic pathways can effectively facilitate reprogramming. For example, phosphoinositide 3-kinase γ (PI3Kγ) is highly active in M2-like TAMs, and its inhibitor IPI-549 (eganelisib) has been shown in preclinical models to reprogram TAMs and synergize with ICIs^224^, ^225^. Epigenetic agents further lock in reprogrammed gene expression profiles by modifying chromatin architecture. Histone deacetylase (HDAC) inhibitors, for instance, increase histone acetylation at M1-associated gene loci, thereby promoting sustained transcriptional activation^226^. Specifically, the selective HDAC6 inhibitor ACY-1215, when combined with PD-1 blockade, synergistically enhances antitumor efficacy in ovarian cancer models, accompanied by a shift of TAM phenotypes toward a pro-inflammatory state^227^ (**Table 1**).

#### 4.1.3 Inhibition of TAM-mediated ECM modification

Building on the finding that TAMs function as key stromal "architects" by secreting crosslinking enzymes to weave highly aligned collagen bundles, inhibiting these ECM-modifying functions represents the most direct strategy to halt tumor mechanical stiffening. Rather than altering TAM abundance or globally reprogramming their phenotype, this approach focuses on targeting key enzymes secreted by TAMs that are responsible for ECM crosslinking, degradation, and remodeling. By disrupting aberrant collagen deposition and structural reorganization, this strategy aims to physically reduce tumor stiffness and improve tissue perfusion.

Targeting the LOX family constitutes a central strategy for suppressing ECM crosslinking. LOX and its homologs, such as LOXL2, are highly expressed in multiple tumor types and promote collagen fiber thickening, alignment, and overall matrix stiffening^228^. TAMs represent a major cellular source of LOX/LOXL2 within the TME^229^. Small-molecule LOXL2 inhibitors, including simtuzumab and PAT-1251, have demonstrated efficacy in preclinical fibrotic and selected tumor models by reducing tissue stiffness, attenuating collagen crosslinking, and suppressing metastasis^230^, ^231^. Furthermore, inhibition of LOX activity can downregulate integrin-FAK/Src signaling in tumor and stromal cells, thereby weakening the mechanically driven positive feedback loop that sustains fibrosis^232^, ^233^. However, phase II/III clinical trials combining simtuzumab with chemotherapy in pancreatic and colorectal cancers failed to improve overall survival^234^, ^235^. These disappointing outcomes highlight the functional complexity and redundancy of the LOX family, as well as the limitations of single-target interventions in advanced, highly fibrotic tumors^236^. In fact, the failure of selective LOXL2 inhibitors is closely associated with the compensatory upregulation of other family members, including LOXL1, LOXL3, and LOXL4. Studies have shown that even when a single isoform is inhibited, they may still collectively maintain collagen crosslinking activity^237^, ^238^. In addition, in established tumors in which the ECM has already undergone extensive crosslinking and stiffening, simply inhibiting further enzymatic activity may be insufficient to reverse the existing physical barrier, because the accumulated matrix remains structurally intact^6^, ^239^. Therefore, current research efforts are shifting toward the development of broader-spectrum LOX family inhibitors, such as dual LOX/LOXL2 inhibitors (e.g., PXS-5153A), or strategies that combine ECM-modulating agents with conventional chemotherapy or immunotherapy, aiming to take advantage of the transient window of matrix remodeling to enhance drug penetration and immune cell infiltration^240^, ^241^.

Modulating the balance between MMPs and their endogenous inhibitors represents another strategy to control pathological ECM degradation and aberrant remodeling^242^, ^243^. TAMs secrete multiple MMPs, including MMP-2, MMP-9, and MMP-12, whose excessive activation and dysregulation contribute to ECM disorganization, enhanced tumor invasion, and the release of pro-tumorigenic factors^244^. Recent studies indicate that MMP-13, through coordinated interactions among CAFs and TAMs in breast cancer, can amplify fibrotic deposition and crosslinking, while under specific spatial and temporal contexts it may also exert antifibrotic or antimetastatic effects^245^, ^246^. However, first-generation broad-spectrum MMP inhibitors, such as marimastat, failed in clinical trials due to limited efficacy and severe musculoskeletal toxicity, largely attributable to their non-selective inhibition of MMPs and consequent disruption of normal tissue homeostasis^247^. Accordingly, next-generation approaches emphasize selective targeting. For instance, the monoclonal antibody DX-2400, which targets membrane-type 1 MMP (MT1-MMP/MMP-14), has demonstrated specific inhibition of tumor growth and metastasis with manageable toxicity in breast cancer models^42^, ^246^. An alternative strategy involves restoring physiological MMP/TIMP balance rather than abolishing enzymatic activity, either by upregulating tissue inhibitor of metalloproteinases-3 (TIMP-3) or by designing TIMP mimetics^248^. In addition, nanoparticle-based systems delivering MMP-9-specific small interfering RNA (siRNA) selectively to TAMs have been developed. In hepatocellular carcinoma models, such approaches achieved localized silencing of MMP-9, effectively suppressing tumor invasion and angiogenesis while avoiding systemic toxicity^249^ (**Table. 1**).

#### 4.1.4 Disruption of TAM mechanosensing pathways

Because extreme mechanical forces in advanced tumors must ultimately be transduced through cellular receptors to drive malignant progression, directly disrupting fundamental mechanosensing pathways (e.g., integrins, FAK, and YAP/TAZ) offers an ultimate strategy to overcome established mechanoresistance, even when TAM abundance is constrained^20^. This constitutes a therapeutic strategy that does not rely on directly altering ECM structure.

Integrins are a major class of mechanosensors that physically connect the ECM to the intracellular cytoskeleton. In TAMs, integrins—particularly αvβ3 and α5β1—undergo clustering upon binding to fibrotic ECM components such as fibronectin and vitronectin, thereby initiating downstream signaling cascades^250^. Small-molecule integrin antagonists and monoclonal antibodies have shown potential in preclinical studies to suppress macrophage-mediated pro-angiogenic and pro-metastatic functions^251^, ^252^. However, cilengitide failed to demonstrate a survival benefit in a phase III clinical trial in glioblastoma, partly due to pathway redundancy and compensatory mechanisms^253^, ^254^. Consequently, current research has shifted toward disrupting the assembly and function of integrin-mediated mechanosignaling complexes rather than simply blocking adhesive interactions. For example, peptides that interfere with the interactions between integrins and cytoskeletal adaptor proteins such as talin and kindlin may more specifically inhibit mechanotransduction while preserving other integrin-dependent biological functions^255^, ^256^.

FAK is a central downstream signaling hub of integrins, responsible for converting mechanical cues into biochemical signals that promote cell survival, proliferation, and migration. In TAMs, FAK activation is closely associated with an immunosuppressive phenotype^257^, ^258^. FAK inhibitors (e.g., defactinib, VS-6063) not only suppress tumor cells in preclinical models of pancreatic cancer and mesothelioma, but also exert significant effects on TAMs^259^, ^260^. Specifically, they reduce the expression of M2 polarization markers in TAMs, decrease the secretion of the pro-fibrotic factor TGF-β, and improve CD8⁺ T-cell infiltration into tumors^261^, ^262^. A phase II clinical trial (NCT02758587) evaluated defactinib in combination with the anti-PD-1 antibody pembrolizumab and chemotherapy in advanced pancreatic cancer, with preliminary results indicating modulation of the immune microenvironment^263^, ^264^. However, because FAK is broadly expressed throughout the body, systemic inhibition may cause off-target toxicity^265^. Therefore, developing strategies that selectively target FAK signaling nodes within macrophages represents an important future direction.

The Hippo pathway effectors YAP/TAZ are key transcriptional co-activators that translate mechanical signals into gene expression programs^266^, ^267^. When TAMs sense a high-stiffness matrix, cytoskeletal tension suppresses the Hippo pathway, leading to YAP/TAZ dephosphorylation and nuclear translocation^267^, ^268^, which in turn drives the expression of a series of pro-fibrotic and pro-proliferative genes, including CTGF, CYR61, and LOX^269^. Accordingly, inhibition of YAP/TAZ represents a critical step in blocking mechanical signal amplification. Verteporfin, an inhibitor of the interaction between YAP/TAZ and TEAD transcription factors^270^, ^271^, has been shown in breast cancer and hepatocellular carcinoma models to suppress TAM-associated tumor progression and fibrosis^272^, ^273^. However, direct pharmacological targeting of transcription factors remains challenging. Alternative approaches include targeting upstream regulatory pathways, such as using the Rho-associated kinase (ROCK) inhibitor fasudil to relax cytoskeletal tension and thereby indirectly suppress YAP/TAZ activation^274^.

Mechanosensitive ion channels provide an intervention point closer to the cell membrane. Channels such as Piezo1 and TRPV4 mediate the sensing of shear stress, stretch, and stiffness changes in multiple innate immune cell types, and activate downstream pathways including MAPK, NF-κB, HIF-1α, and YAP/TAZ through Ca²⁺ influx^275^, ^276^. In the tumor context, selectively blocking Piezo/TRPV channels expressed by TAMs using small-molecule inhibitors or nanoparticle-based delivery systems may attenuate their aberrant responses to elevated interstitial pressure and fluid shear stress, thereby suppressing HIF-1α-driven expression of pro-fibrotic factors such as SPP1 and LOX^277^. It must be noted, however, that mechanosensing pathways are highly conserved across cell types and play essential roles in tissue homeostasis^278^; thus, their systemic inhibition may result in widespread toxicity. Future research should therefore focus on developing interventions with greater cell-type specificity or enabling precise local delivery (**Table 1**).

Taken together, the reciprocal interaction between TAMs and the mechanical microenvironment is not static but evolves dynamically during tumor progression, forming a progressively reinforced biomechanical-immunological feedback system. In this context, presenting TAM-targeted therapeutic strategies as independent or parallel options may obscure their potential stage-specific relevance in clinical settings. To enhance translational applicability, it is therefore valuable to conceptualize these strategies within a longitudinal intervention framework aligned with the mechanical evolution of the tumor stroma.

During the early phase of tumor development, when inflammation-driven matrix remodeling and active monocyte recruitment predominate and the stromal barrier remains relatively permissive^279^, ^280^, interventions that limit TAM accumulation or reprogram their highly plastic phenotypes may effectively prevent the initial establishment of pro-fibrotic niches. As tumors progress toward an intermediate stage characterized by active ECM crosslinking and progressive tissue stiffening, TAMs cooperate with stromal cells to promote dense collagen deposition and matrix remodeling^47^. At this stage, targeting TAM-derived ECM-modifying enzymes, such as LOX and MMPs, may help restrain the rapid escalation of stromal rigidity. In more advanced tumors, where highly crosslinked matrices generate sustained mechanical stress that stabilizes immunosuppressive TAM programs^11^, ^20^, therapeutic strategies may need to shift toward disrupting core mechanotransduction pathways—such as integrin-FAK-YAP signaling—often in combination with matrix-modulating approaches to overcome established mechano-immunological resistance.

Despite the diverse strategies targeting TAMs at the cellular and molecular levels, these approaches are often limited by insufficient spatial precision, transient effects, and the inability to directly reconstitute the physical architecture of the tumor microenvironment. As tumor progression is governed not only by cellular behaviors but also by dynamically evolving biomechanical contexts, there is an increasing need for strategies that can actively and precisely modulate the physical and immunological properties of the TME in situ. Indeed, given the inherent limitations of single-agent TAM-targeted strategies-such as off-target effects, incomplete mechanical remodeling, and compensatory resistance-biomaterial-based mechano-immunological engineering offers a novel paradigm for the spatiotemporally precise modulation of the tumor microenvironment.

### 4.2 Mechanical immunoengineering and biomaterial-based strategies

Mechanical immunoengineering aims to exploit rationally designable biomaterials to actively create or mimic specific physicochemical microenvironments, thereby enabling precise, in situ regulation of TAMs and other immune cells. This approach represents a transformative strategy for overcoming intrinsic tumor mechanical barriers and implementing “active softening” of the TME.

#### 4.2.1 Mechanically programmable hydrogels and scaffolds

Mechanically programmable hydrogels or scaffolds enable in situ recruitment and controllable phenotypic reprogramming of TAMs by precisely regulating matrix stiffness, degradation kinetics, and three-dimensional architecture, without the need for systemic administration. This strategy is a representative paradigm within the field of mechanical immunoengineering.

One major approach focuses on the development of injectable hydrogels with spatiotemporally controllable immunomodulatory functions. These materials typically exhibit favorable injectability and in situ gelation properties, allowing them to fill irregular tissue cavities and form mechanically tunable network structures through chemical or physical crosslinking, thereby serving as platforms for local sustained drug release and mechanical signal presentation. For example, an injectable hydrogel composite system based on carbon dots and proteins (TTF-L-C) has been used for multistage regulation of TAMs. In this system, the carbon dot component mediates chemotactic effects by upregulating Ctnnd1 expression, promoting selective macrophage accumulation within the gel. Subsequently, lipopolysaccharide encapsulated in the hydrogel locally activates macrophages and drives their polarization toward an M1-like phenotype, while concurrent administration of a PD-L1 blockade prevents potential compensatory upregulation of PD-L1 following reprogramming. As the hydrogel gradually degrades, these “trained” M1-like macrophages are continuously released into the tumor site, thereby promoting dendritic cell maturation and T-cell activation. In both primary and recurrent 4T1 breast cancer models, this strategy significantly enhanced immunotherapeutic efficacy and demonstrated favorable safety profiles^300^. This design elevates hydrogels from passive carriers to in situ activation platforms capable of actively modulating the local immune microenvironment. Another representative study developed a radiation-responsive self-assembling peptide hydrogel that undergoes conformational changes upon radiotherapy, enabling on-demand release of TLR7/8 agonists^301^. This allows precise temporal coordination between radiotherapy-induced immunogenic cell death (ICD) and TAM reprogramming, thereby markedly enhancing antitumor efficacy when combined with PD-1 blockade^302^. Collectively, these studies demonstrate that coupling material degradation kinetics with drug release dynamics enables single-intervention yet long-term regulation of TAMs, achieving temporally precise therapeutic control.

Another class of strategies focuses on the development of implantable scaffold systems that integrate both mechanical support and immunomodulatory functions, particularly suited for clinical scenarios combining postoperative tumor control with tissue regeneration. These scaffolds not only provide macroscopic mechanical support to defect sites, but their porous three-dimensional structures also offer an ideal matrix for regulating cell behavior and local drug delivery. For example, in postoperative bone tumor models, researchers constructed 3D-printed calcium phosphate scaffolds loaded with the CSF-1R inhibitor BLZ945. During the early post-implantation phase, the scaffold functions as a local drug reservoir, continuously releasing the inhibitor to selectively block the CSF-1/CSF-1R axis, thereby effectively depleting TAMs and suppressing pro-tumorigenic M2-like polarization to alleviate local immunosuppression. Meanwhile, the scaffold structure itself serves as a physical barrier. At later stages, as the scaffold surface gradually degrades, its internal architecture guides bone tissue regeneration^303^. Similarly, a self-healing hydrogel based on RADA peptides capable of loading the CaMKII inhibitor KN93 has been shown, in models such as malignant ascites, to induce immunogenic tumor cell death and directly reprogram M2-like TAMs (via inhibition of their highly expressed CaMKII) following a single intraperitoneal injection, thereby synergistically enhancing antitumor immune responses^304^. Such multifunctional integrated systems, combining chemotherapy, TAM reprogramming, and immune activation, fully illustrate the substantial potential of biomaterials as active therapeutic platforms.

#### 4.2.2 TAM-targeted biomaterial delivery systems

Material-based delivery systems targeting TAMs constitute another central pillar of mechanobiological immunoengineering. Unlike hydrogels or scaffolds that provide fixed mechanical frameworks, these systems achieve selective recognition and intervention of TAMs and their monocyte precursors by precisely tuning the physicochemical properties of nanocarriers and their surface ligands. Relying on systemic or intracavitary administration, such platforms enable immune modulation across broader spatial scales of the TME and can act synergistically with immune checkpoint blockade, chemotherapy, or radiotherapy.

In terms of targeting strategies, current designs predominantly exploit receptors or surface molecules that are highly expressed on pro-tumoral TAM subsets, including CD163, CD206/MRC1, Siglecs, and MGL^305^, ^306^. Selective uptake and intratumoral enrichment are achieved through antibodies, short peptides, or glycan-based modifications^305^. These receptors not only mark immunosuppressive phenotypes but are also functionally linked to matrix-degrading enzymes and pro-angiogenic factors, thereby directly participating in tissue remodeling and alterations of mechanical properties. For example, polymeric prodrug nanoparticles loaded with doxorubicin and functionalized with anti-CD163 monoclonal antibodies undergo disassembly within acidic endosomal compartments, enabling preferential drug release in CD163⁺ M2-like TAMs and enhanced tumor suppression^305^, ^307^. Similarly, liposomes engineered based on the sialic acid-Siglec axis increase uptake by circulating monocytes and TAMs via surface-grafted sialic acids, thereby attenuating the continuous recruitment of immunosuppressive myeloid cells into tumors and slowing cell influx associated with M2 polarization and tissue remodeling^306^, ^308^. Mannose- or galactose-modified albumin and high-density lipoprotein-mimetic nanoparticles selectively bind CD206/MGL-positive TAMs, enriching clodronate, Toll-like receptor (TLR) agonists, or small-molecule immunomodulators within this population to enable selective depletion or inflammatory phenotype conversion^307^, ^309^, ^310^.

In recent years, the delivery of informational biomolecules—such as nucleic acids and proteins—to rewire TAM signaling networks and transcriptional programs has emerged as a major direction. These strategies typically target key nodes involved in metabolism, epigenetic regulation, and cytoskeletal remodeling, thereby synchronously reprogramming immune and mechanical behaviors^311^. Multiple studies have demonstrated that ligand-modified nanoparticles encapsulating mRNAs encoding interferon regulatory factor 5 (IRF5) and IκB kinase β (IKKβ) can stably drive TAM polarization toward an M1-like state in ovarian cancer, melanoma, and glioblastoma models, accompanied by increased CD8⁺ T-cell infiltration and marked tumor regression^312^. Such M1-like TAMs generally secrete fewer pro-fibrotic and pro-angiogenic factors, thereby restraining collagen deposition and aberrant vascular expansion and alleviating stromal stiffening^48^, ^313^. CRISPR/Cas9-based delivery platforms further enable gene-level intervention of critical regulators such as PI3Kγ, a key modulator of TAM metabolism and migration that is closely associated with cytoskeletal reorganization and chemotactic motility^314^, ^315^. In murine solid tumor models, co-delivery of Cas9 complexes targeting Pik3cg with CpG-rich bacterial-derived nanovesicles simultaneously blocks PI3Kγ signaling and activates TLR9. These specialized vesicles markedly reduce the proportion of immunosuppressive TAM subsets and, when combined with PD-1 blockade, convert immunologically cold tumors into states highly responsive to immunotherapy^309^, ^316^. In addition, mannose-decorated hierarchical micelles have been used to deliver TLR7/8 agonists such as R848 to TAMs in gliomas, enabling M2-to-M1 reprogramming across the blood-brain barrier and reversing resistance to temozolomide^317^, ^318^. Activated M1-like TAMs in gliomas further modulate ECM secretion by stromal and astrocytic cells, improving local tissue compliance and drug diffusion^319^.

Representative nanomedicine-based combination strategies that integrate immunosuppressive microenvironment remodeling with ICD induction typically operate through two synergistic mechanisms. First, TAM-targeting nanocarriers are used to deliver polarization cues that directly reprogram pro-tumorigenic M2-TAMs into pro-inflammatory M1-TAMs, thereby suppressing tumor growth and limiting metastatic dissemination^154^, ^320^. Multiple platforms, including M2pep-modified ferritin nanocages delivering CpG(311), HA-PEI nanoparticles loaded with miR-125b^321^, and HA-mannose-modified nanocapsules co-encapsulating poly(I:C) and R848^291^, have consistently demonstrated robust M1 polarization and significant inhibition of primary and metastatic tumor progression across diverse solid tumor models. Second, chemotherapeutic agents or photothermal/photosensitizing components are incorporated to exploit ICD-mediated immune amplification. Nanocarrier-delivered anthracyclines such as doxorubicin (DOX), as well as taxanes, efficiently induce hallmark ICD signals, including DAMP exposure and HMGB1 release, leading to enhanced dendritic cell activation, increased CD8⁺ T-cell infiltration, and elevated effector cytokine production^322^, ^323^. In parallel, these systems frequently reduce the infiltration of Tregs, MDSCs, and TAMs, while suppressing angiogenic programs, resulting in pronounced tumor regression and metastasis inhibition^324^. Importantly, combining TAM-targeted immunostimulatory agents (e.g., R848) with conventional chemotherapy within coordinated nanodelivery systems further amplifies these effects, effectively converting “cold” tumors into immunotherapy-responsive states and enhancing the efficacy of radiochemotherapy or PD-1/PD-L1 blockade^302^, ^310^. Collectively, these findings underscore that coupling nanodelivery, TAM reprogramming, and ICD induction represents a streamlined and broadly applicable strategy for synergistically potentiating chemo-immunotherapy in solid tumors.

Overall, TAM-targeted material delivery systems achieve indirect yet pivotal regulation of tumor immune and mechanical microenvironments through systematic design of targeting recognition, cargo loading, environmental responsiveness, and effector cell reprogramming. Future integration of mechano-responsive structural units, TAM-targeting modules, and immune checkpoint regulatory elements within a single platform may enable more compact therapeutic strategies capable of coordinately reshaping tumor mechanical and immune landscapes** (Table 2)**.

While biomaterial-based and mechanical immunoengineering strategies provide powerful platforms for localized and programmable modulation of TAMs and tumor mechanics, their therapeutic efficacy as standalone interventions may still be constrained by the multifactorial nature of tumor progression and resistance. Given that TAM-driven immunosuppression and mechanical remodeling are tightly intertwined with angiogenesis, metabolic reprogramming, and treatment-induced adaptation, integrating TAM-targeted approaches with established therapeutic modalities has become a critical direction for achieving more durable and systemic antitumor effects.

### 4.3 Combination therapeutic strategies

As mechanistic insights into TAM biology and the efficacy of single-agent interventions have matured, combination therapeutic strategies have emerged as a critical avenue for improving treatment outcomes, particularly for remodeling the immunosuppressive and mechanically aberrant TME. TAMs contribute to tumor progression through dual mechanisms: on the one hand, they promote angiogenesis, ECM remodeling, and interstitial pressure elevation, thereby establishing a rigid, poorly perfused physical barrier; on the other hand, they sustain profound immune suppression. Accordingly, integrating TAM depletion or reprogramming strategies with ICIs, anti-angiogenic or anti-fibrotic therapies, and radiochemotherapy holds promise not only for overcoming immune tolerance but also for reshaping the mechanical microenvironment, ultimately restoring effective antitumor immune surveillance.

#### 4.3.1 Combination with ICIs

Among combination paradigms, the integration of TAM-targeting strategies with ICIs is supported by the most substantial body of clinical and preclinical evidence. Multiple studies have demonstrated that M2-like TAMs are a major driver of both primary resistance and acquired refractoriness to PD-1/PD-L1 and cytotoxic T-lymphocyte-associated protein 4 (CTLA-4) blockade. M2-like TAMs suppress effector T-cell activity through high expression of PD-L1, Arg1, and IDO, as well as through secretion of immunosuppressive cytokines such as IL-10 and TGF-β. In parallel, TAM-derived VEGF, MMPs, and chemokines sustain abnormal vasculature, dense ECM deposition, and restricted immune cell infiltration, collectively reinforcing an immunosuppressive and mechanically hostile tumor niche.

On this basis, combining TAM-targeted interventions with ICIs can generate synergistic effects at both the immune and tissue-structural levels. On the one hand, reducing the abundance and suppressive functions of M2-like TAMs through CSF-1R inhibition, blockade of the CCR2/CCL2 axis, or PI3Kγ inhibitors attenuates their antagonistic effects on ICIs^197^, ^205^, ^299^. On the other hand, reprogramming TAMs toward an M1-like phenotype using TLR or CD40 agonists, small-molecule immunomodulators, or nanodelivery platforms enhances antigen-presenting capacity and pro-inflammatory cytokine production, while promoting the recruitment and functional activity of dendritic cells and CD8⁺ T cells within tumors^292^, ^333^.

In the phase II MARIO-3 trial in metastatic triple-negative breast cancer, the PI3Kγ inhibitor eganelisib combined with an anti-PD-L1 antibody and nab-paclitaxel not only induced TAM reprogramming and T-cell activation, but was also accompanied by downregulation of ECM-related transcriptional signatures and reorganization of vascular-immune gene programs, suggesting that TAM-targeted therapy combined with ICIs and chemotherapy may concurrently improve immune and stromal structural features(NCT03961698)^334^. Similarly, in melanoma and other animal models, interventions targeting macrophage-associated receptors such as MARCO in combination with CTLA-4 blockade shifted chemokine profiles from an M2-biased immunosuppressive pattern toward one favoring M1-like inflammatory infiltration, thereby significantly increasing effector T-cell entry into tumors and enhancing tumor burden control^335^. Taken together, current evidence indicates that TAM-targeted strategies combined with ICIs not only potentiate antitumor immune responses, but may also optimize the immune and mechanical properties of tumors by alleviating abnormal vascular leakage and excessive ECM deposition, improving local perfusion, and facilitating immune cell migration within dense stromal regions.

#### 4.3.2 Combination with anti-angiogenic or anti-fibrotic therapies

The combination of TAM-targeted strategies with anti-angiogenic or anti-fibrotic therapies focuses on modulating the mechanical state of the TME at the interface of vasculature, ECM, and immune cells. Classical VEGF/VEGFR inhibitors, when administered within an appropriate dosing window, can partially normalize vascular structure and function, thereby improving perfusion and enhancing the delivery of therapeutics and immune cells. However, prolonged or excessive angiogenesis inhibition readily induces secondary hypoxia, which drives the accumulation of M2-like TAMs and activation of pro-fibrotic programs, accelerates ECM deposition, and ultimately reinforces tissue stiffness and immunosuppression^336^.

Recent perspectives emphasize that therapeutic strategies simultaneously targeting angiogenesis and TAMs can more effectively disrupt these pathological feedback loops. On the one hand, inhibiting TAM-derived VEGF, PDGF, MMPs, LOX, or their upstream regulatory pathways (such as HIF-1α and PI3Kγ signaling) may reduce aberrant angiogenesis and collagen crosslinking, thereby alleviating ECM densification and elevated interstitial pressure at their source^314^, ^337^. On the other hand, combining anti-VEGF/VEGFR agents or multi-target tyrosine kinase inhibitors with TAM reprogramming approaches can, while improving vascular morphology and reducing leakage and tissue edema, attenuate pro-fibrotic interactions between M2-like TAMs and CAFs, leading to decreased collagen deposition and matrix crosslinking^338^, ^339^.

For example, the heme oxygenase-1 (HO-1) inhibitor KCL-HO-1i suppresses LYVE-1⁺ perivascular TAMs and, when combined with cytotoxic chemotherapy, significantly enhances therapeutic responses in chemotherapy-resistant breast cancer and sarcoma models. This is accompanied by a shift of tumors from an immune-excluded state toward an inflamed microenvironment enriched in CD8⁺ T cells, together with vascular structural and functional parameters approaching a more physiological state^340^. Clinical practice in hepatocellular carcinoma, renal cell carcinoma, and endometrial cancer has demonstrated that combining anti-angiogenic therapy with ICIs improves objective response rates and survival outcomes. Building on this foundation, further incorporation of TAM-targeted interventions may reduce residual M2 TAM-mediated mechanical and immune resistance, thereby enabling more durable microenvironmental remodeling.

#### 4.3.3 Combination with radiotherapy and chemotherapy

The combination of TAM modulation with radiotherapy and chemotherapy reflects an evolution of therapeutic strategies that incorporate microenvironmental regulation on the basis of conventional local or systemic cytotoxic treatments. Various chemotherapeutic agents and radiotherapy can induce ICD of tumor cells, leading to the release of tumor-associated antigens and damage-associated molecular patterns (DAMPs), thereby providing an antigenic source for the initiation and amplification of antitumor T-cell responses^341^. However, these treatments can also promote a shift of TAMs toward pro-angiogenic and pro-fibrotic M2-like phenotypes through vascular damage and inflammation-associated cytokine release, resulting in ECM remodeling and aberrant vascular repair^342^. Over certain temporal scales, this process gives rise to tolerance that encompasses both mechanical and immune dimensions.

Integrating TAM depletion or reprogramming interventions into radiotherapy or chemotherapy can re-direct tissue repair pathways while maintaining or enhancing cytotoxic efficacy. On the one hand, delivery platforms such as liposomal or polymeric nanoparticles can co-load ICD inducers, including doxorubicin, together with TAM-depleting or reprogramming agents, enabling a single therapeutic regimen to simultaneously trigger ICD within tumor cells and reduce M2-like TAM abundance and regulatory T-cell recruitment in the microenvironment. This restores local pro-inflammatory cytokine profiles and amplifies ICD-driven T-cell responses and memory formation. In 4T1 breast cancer mouse models, such “dual-targeting” designs significantly reduce M2-like TAMs, increase M1-like TAMs and effector T-cell infiltration, and suppress distant lung metastasis^324^, ^343^.

On the other hand, radiotherapy alters tumor-region hemodynamics and matrix architecture through endothelial injury and parenchymal inflammation^344^, during which TAMs participate in post-irradiation vascular repair and fibrosis and critically determine the modes of inflammation resolution and necrotic tissue clearance^345^. Available evidence indicates that adding CD40 or Toll-like receptor (TLR) agonists to local radiotherapy activates TAMs and drives their polarization toward M1-like states, thereby enhancing both local and abscopal antitumor responses and improving long-term tumor control^216^. If further combined with small-molecule or nanomedicine-based inhibitors targeting LOX, MMPs, or cathepsins, this strategy may restrain excessive ECM crosslinking and fibrosis during the early phase of radiation-induced tissue remodeling, preventing sustained loss of stromal compliance and restricted drug diffusion.

#### 4.3.4 Additional combination strategies

Building on these approaches, integrating TAM-targeted nanoplatforms with the above combination strategies provides a feasible route for multilevel interventions spanning cellular, tissue, and organ scales. Recent studies have proposed that ligand-modified nanoparticles or vesicular systems can selectively deliver TLR/CD40 agonists, PI3Kγ inhibitors, or CRISPR/Cas9 components to specific TAM subsets, thereby enhancing antitumor immune responses while simultaneously influencing how these cells regulate ECM-producing cells and vascular endothelial cells. Ultimately, such strategies alter collagen fiber organization, vascular permeability, and local tissue compliance.

For example, in glioblastoma models, TAM-targeted TLR7/8 agonists are able to cross the blood-brain barrier and induce the conversion of M2-like TAMs toward M1-like phenotypes, thereby modifying their regulation of astrocytes and ECM components and improving the mechanical properties of brain tissue and drug diffusion^346^. When combined with ICIs, anti-angiogenic agents, or temozolomide, these platforms can simultaneously optimize immune activity and physical barrier characteristics within intracranial microenvironments characterized by high stiffness and restricted geometric space, leading to improved overall therapeutic responses in refractory tumors.

**Figure 8:**
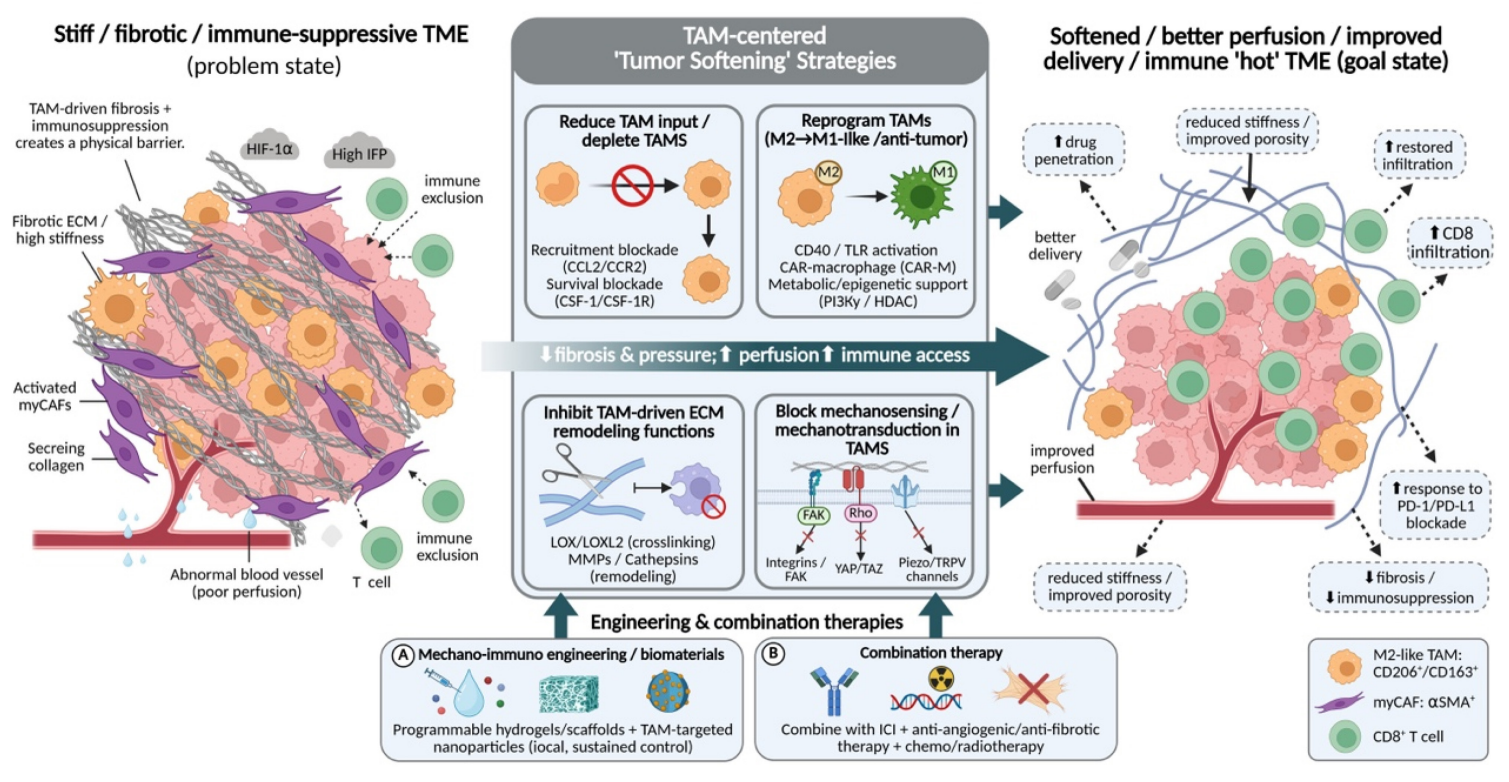
TAM-centered tumor softening strategies to recondition a stiff, fibrotic, immune-suppressive TME. Left, TAM-myCAF-driven collagen deposition elevates ECM stiffness and IFP, induces HIF-1α-associated hypoxia, and sustains abnormal vessels with poor perfusion, leading to immune exclusion. Center, intervention axes that reduce fibrosis/pressure and restore perfusion/immune access: (i) limit TAM input or deplete TAMs (CCL2-CCR2; CSF-1/CSF-1R), (ii) reprogram TAMs toward anti-tumor states (CD40/TLR; CAR-M; PI3KPPAR/HDAC), (iii) inhibit TAM-mediated ECM remodeling (LOX/LOXL2; MMPs/cathepsins), and (iv) block TAM mechanosensing/mechanotransduction (integrin/FAK, Rho, YAP/TAZ, Piezo/TRPV). Right, reduced stiffness improves porosity, drug penetration, CD8⁺ infiltration, and response to PD-1/PD-L1 blockade.

In summary, TAM-centered combination strategies are evolving from purely immunological synergy toward integrated regulatory paradigms that jointly address immune and mechanical dimensions. Whether combined with ICIs, anti-angiogenic/anti-fibrotic therapies, or radiotherapy, chemotherapy, and nanodelivery platforms, these approaches share a common objective: to selectively deplete or reprogram pro-tumorigenic TAMs, thereby relieving physical barriers to drug delivery and immune cell infiltration while attenuating TAM-driven immunosuppressive networks (**Fig. 8**).

### 4.4 Comparative analysis and key challenges in clinical translation

Although the aforementioned therapeutic strategies have demonstrated encouraging efficacy in preclinical models, their translation into clinical practice remains limited by the multifactorial and adaptive nature of tumor progression. A critical evaluation indicates that, despite distinct mechanistic targets, these approaches share fundamental constraints arising from biological redundancy, spatial heterogeneity, and systemic complexity.

From a comparative perspective, TAM-targeted strategies differ across several key dimensions, including targeting specificity, spatiotemporal controllability, durability, and translational feasibility. TAM depletion strategies (e.g., CSF-1R or CCL2-CCR2 axis blockade) directly reduce immunosuppressive populations within the tumor microenvironment; however, their clinical efficacy is often limited by immune plasticity and functional compensation, including the recruitment of alternative suppressive myeloid populations such as PMN-MDSCs, as well as potential depletion of tissue-resident macrophages, leading to systemic toxicity^347^, ^348^.

In contrast, TAM reprogramming strategies (e.g., CD40/TLR agonists or CAR-M approaches) preserve macrophage populations while redirecting their functional states toward antitumor activity and matrix remodeling. However, these effects are frequently transient, as the persistent mechanical and metabolic pressures within the tumor microenvironment can drive phenotypic reversion toward pro-tumoral states in the absence of sustained stimulation or engineered retention systems^265^, ^349^. Mechanobiology-targeted strategies (e.g., LOX, MMP, FAK, or integrin inhibition) directly address the physical basis of tumor progression and stromal stiffening; nevertheless, their clinical application is challenged by pathway redundancy and systemic pleiotropy, given the essential roles of these signaling axes in normal tissue homeostasis, often resulting in dose-limiting toxicities prior to achieving effective intratumoral exposure^350^.

Collectively, these approaches exhibit distinct but complementary limitations: depletion strategies are limited by compensatory immune responses and toxicity; reprogramming strategies are constrained by temporal instability; and mechanobiology-targeted interventions face challenges related to redundancy and safety.

Beyond target-specific limitations, three overarching challenges further constrain clinical translation. First, the physical delivery paradox: dense, highly crosslinked collagen networks and elevated interstitial fluid pressure significantly restrict intratumoral penetration, particularly limiting access to poorly vascularized regions enriched in M2-like TAMs and cancer-associated fibroblasts^351^. Second, the lack of mechanobiology-informed biomarkers: current clinical stratification relies primarily on disease stage rather than tumor mechanical properties or TAM functional states, underscoring the need for validated non-invasive indicators such as ECM-derived circulating fragments, mechanotransduction-associated gene signatures, or elastography-based parameters^59^. Third, the context-dependent duality of ECM remodeling: while matrix deposition restricts tumor expansion and immune infiltration in certain contexts, excessive or untimely degradation may paradoxically facilitate tumor dissemination and metastatic progression^342^.

Taken together, these limitations highlight the intrinsic constraints of single-modality approaches in the tumor mechano-immune ecosystem. Although emerging biomaterial-based and combination strategies offer improved spatiotemporal control and therapeutic synergy, they also introduce additional translational barriers, including manufacturing complexity, scalability, immunogenicity risks, and challenges in dose optimization and mechanistic deconvolution. Accordingly, the rational integration of complementary strategies, supported by mechanistic biomarkers and multi-omics monitoring, represents a promising yet still technically demanding direction for future clinical translation.

## 5. Challenges and future directions

TAMs serve as the central hub linking the tumor immune microenvironment and mechanical microenvironment^69^, ^352^. By shaping ECM stiffness, fiber alignment, and interstitial pressure, TAMs drive tumor progression and therapeutic resistance. Simultaneously, TAMs themselves are reciprocally regulated by mechanical signals, forming a vicious cycle^13^. Therefore, TAMs represent a key therapeutic target, and strategies such as depletion, reprogramming, the blockade of mechanical sensing, and material engineering aim to "soften" the tumor, thereby improving drug delivery and enhancing immune infiltration.

Despite the promising therapeutic strategy of targeting TAMs to remodel the tumor mechanical microenvironment, this field still faces a series of profound and interconnected challenges. Firstly, both the tumor mechanical microenvironment and TAM populations exhibit high spatiotemporal heterogeneity, which is the primary obstacle to precise intervention. Spatially, distinct tumor regions differ significantly in ECM stiffness, architecture, and immune composition^59^. Recent studies have revealed that profibrotic SPP1^+^ TAM subsets and proangiogenic TIE2^+^ TAM subsets occupy completely distinct tumoral regions, each colocalizing with specific mechanical characteristics^353^. Temporally, both the mechanical microenvironment and TAM phenotypes undergo dynamic evolution with tumor progression and therapeutic intervention^354^. This heterogeneity implies that generalized TAM-targeting strategies may fail to eliminate key pathogenic subsets and may disrupt macrophages with antitumor potential. Therefore, a core future challenge lies in developing technologies capable of real-time identification and targeting of specific functional TAM subsets. This may require integrating strategies such as activatable antibody-drug conjugates (ADCs), chimeric antigen receptor macrophage (CAR-M) design based on specific activation markers on TAM surfaces, or smart material delivery systems responsive to local mechanical properties to achieve precise intervention.

Current experimental studies on the interactions between immune cells/macrophages and the mechanical microenvironment are still largely based on mouse tumor models or static 2D/3D in vitro systems, whereas these models exhibit systematic deviations from human tumors in key parameters such as matrix stiffness, collagen crosslinking, and interstitial fluid mechanics^59^, ^355^. For example, elastography measurements show that human tumors exhibit substantially higher stiffness than corresponding mouse models^356^. Conventional 2D plastic culture completely lacks three-dimensional ECM topology, while commonly used hydrogels are limited in stiffness range, reproducibility, and architectural control, making it difficult to recapitulate the complex, mature collagen networks and their dynamic stiffening processes observed in human solid tumor^357^. In recent years, tumor-/organ-on-chip platforms integrating patient-derived organoids with CAFs, endothelial cells, and immune cells, together with perfusable microvasculature and controllable interstitial flow, have enabled the establishment of vascular perfusion, pressure gradients, and drug transport environments that more closely resemble in vivo conditions at the microscale. These systems have already been applied to evaluate drug penetration and efficacy within 3D matrices, providing tools with greater translational potential for dissecting and modulating TAM-associated mechanobiological niches^358^.

Addressing these limitations increasingly relies on emerging advanced technologies that enable high-resolution, dynamic, and systems-level interrogation of tumor mechanobiology. Recent advances in spatial transcriptomics allow mapping of TAM subpopulations within intact tumor architectures, linking integrin-associated signaling states with local mechanical niches and immune landscapes^359^. In parallel, in vivo mechanical imaging techniques enable dynamic visualization of mechanical properties in living tissues, bridging static measurements with the spatiotemporal evolution of mechanotransductive signaling^360^, ^361^. Furthermore, machine learning-based frameworks integrate multi-omics, spatial, and biomechanical data to reconstruct mechanotransductive networks and predict TAM behavior under varying conditions^362^, ^363^. Collectively, these approaches provide a foundation for translating mechanistic insights into quantitatively testable and clinically actionable models.

In the future, it is necessary to vigorously develop and standardize these advanced models that can accurately simulate the mechanical properties of human tumors, including dynamic stiffness-tunable hydrogels, organoids integrated with biomechanical sensors, and bioreactor systems capable of applying cyclic solid stress or interstitial pressure within physiological ranges. These models will more reliably predict the efficacy of drugs in softening tumor mechanical barriers and reprogramming TAMs in humans.

Translating laboratory findings into clinically applicable therapies remains a long and arduous journey. First, identifying predictive biomarkers is crucial. We need to clarify which patients are most likely to benefit from TAM-targeted "softening" strategies. Peripheral blood monocyte subsets, circulating collagen metabolites, or imaging-derived mechanical parameters may serve as potential biomarkers. Second, treatment timing and sequencing need optimization. Key questions include: At which stage, before, during, or after immune checkpoint inhibitor therapy, is TAM-targeted intervention most effective? These questions require exploration in well-designed clinical trials. Recent clinical trials have provided preliminary clues. For example, early-phase clinical trials of CD40 agonists combined with chemotherapy in pancreatic cancer have shown changes in the immune and stromal microenvironments, but the efficacy awaits verification in larger cohorts^213^. In the future, more clinical trials incorporating mechanical microenvironment parameters as endpoints are needed—for instance, using serial imaging to assess changes in tumor stiffness before and after treatment, and correlating these changes with pathological responses and survival outcomes.

Targeting TAMs to remodel the tumor mechanical microenvironment is a cutting-edge field full of opportunities and challenges. It requires us to go beyond the traditional biochemical perspective and recognize mechanics as a therapeutic dimension equally important to immunity. With the deepening understanding of TAMs as "mechano-immune hubs" and the emergence of new technologies and strategies, we have reason to anticipate that interventions targeting the tumor mechanical microenvironment will evolve from auxiliary approaches to pillar strategies—providing a novel breakthrough for improving drug delivery, overcoming immune resistance, and ultimately combating solid tumors.

## Figures and Tables

**Table 1:** Summary of therapeutic strategies targeting TAMs for tumor mechanical softening

Drug	Target	Core Mechanism	Ref.
i. TAMs Depletion and Inhibition of Monocyte Recruitment			
FF-10101	Covalent small-molecule inhibitor (FLT3 / CSF-1R)	Sustained inhibition of CSF-1R to deplete immunosuppressive TAMs.	205
BLZ945	Small-molecule inhibitor (CSF-1R)	Inhibits CSF-1R to reduce TAMs/microglia and attenuate stromal support in brain metastases.	281
PLX5622	Small-molecule inhibitor (CSF-1R)	Depletes a subset of CSF-1R-dependent TAMs to delay tumor progression.	202
GW2580	Small-molecule inhibitor (CSF-1R)	Reduces infiltration of profibrotic (Ly6C⁺ M2-like) macrophages to alleviate fibrosis.	282
Unnamed c-FMS inhibitor	Small-molecule inhibitor (CSF-1R)	Blocks CSF-1R to reduce TAM infiltration and CAF-rich dense stroma, partially restoring tissue structure.	283
PXB17	CSF-1R	Next-gen inhibition; depletes M2-TAMs and stabilizes T-cells.	197
PF-04136309	CCR2	Blocks monocyte recruitment; weakens fibrotic stromal support.	192
Anti-OPN/αvβ3	OPN-αvβ3	Spatial targeting; selectively suppresses pro-fibrotic TAM subsets.	208
Anti-GM-CSF mAb	Neutralizing antibody (GM-CSF)	Blocks the GM-CSF-bone marrow axis to inhibit monopoiesis and TAM/MDSC replenishment.	284
ii. TAMs Reprogramming			
Anti-CD40 agonist (PDA, mouse)	Agonistic antibody (CD40)	Activates myeloid/DC cells to convert "cold" tumors into T-cell-sensitive state.	285
Anti-CD40 agonist (PDA + T-cell therapy)	Agonistic antibody (CD40)	Reprograms TAMs towards an inflammatory phenotype to enhance engineered T-cell longevity (superior to depletion).	286
Anti-CD40 + Anti-PD-1	Antibody combination (CD40 + PD-1)	CD40 activation reprograms TAMs/DCs; PD-1 blockade synergizes to enhance cytotoxic T-cell response.	287-289
FLT3L + Anti-CD40	Growth factor + Agonistic antibody (Flt3L + CD40)	Restores and activates cDCs to trigger a type 1 immune response in fibrotic tumors.	290
poly(I:C) + R848	TLR agonist combination (TLR3 + TLR7/8)	Synergistically reprograms M2-like macrophages to M1-like, cytotoxic effectors.	291
R848 + poly(I:C) (lung cancer)	TLR agonist combination (TLR7/8 + TLR3)	Drives TAM repolarization towards M1 phenotype and promotes T-cell recruitment.	292
1V270	Small-molecule TLR7 agonist	Intratumoral injection increases M1/M2 ratio in TAMs and enhances antigen presentation.	293
Lipo-MP-LPS	Liposome-encapsulated LPS analog (TLR4 agonist)	Induces M2→M1 conversion systemically, promoting a pro-inflammatory TME.	294
AZD2796 (anti-LILRB2)	Antibody (LILRB2)	Reprograms TAMs towards an immunostimulatory phenotype by blocking the myeloid checkpoint LILRB2.	295
iii. Inhibition of TAM-Mediated ECM Modification			
BAPN	Small-molecule LOX inhibitor	Inhibits collagen cross-linking to reduce tumor stiffness and improve T-cell migration.	59
Anti-LOXL2 neutralizing antibody	Antibody (targets extracellular LOXL2)	Inhibits collagen stabilization and promotes macrophage-mediated "on-fiber" collagenolysis.	296
PXS-5153A	LOX/LOXL2	Dual inhibition; blocks collagen crosslinking and reduces stiffness.	234, 241
DX-2400	MT1-MMP	Selective MMP block; inhibits pathological matrix remodeling.	246
CAR-147 macrophages	Cellular therapy (CAR-M)	HER2-targeted CAR-M cells are engineered to upregulate MMPs for localized, precise ECM degradation.	49
iv. Disruption of TAM Mechanosensing Pathways			
VS-4718	Small-molecule FAK inhibitor	Inhibits integrin-FAK signaling driven by matrix stiffness, reducing YAP/TAZ activity.	262
Defactinib, GSK2256098	Small-molecule FAK inhibitors	Block FAK-mediated adhesion, migration, and stiffness-sensing pathways.	297
iTEAD	Small-molecule TEAD inhibitor	Blocks the terminal transcriptional output (YAP/TAZ-TEAD) of ECM stiffness signaling.	298
Verteporfin	YAP/TAZ	Interrupts transcriptional decoding of extracellular mechanical cues.	270, 271
Fasudil	ROCK	Relaxes cytoskeletal tension; suppresses YAP-mediated activation.	274
TB511	Peptide-drug conjugate (targets activated CD18)	Targets and deletes M2-TAMs with high conformational activation of integrin CD18.	299

**Table 2:** Advanced Biomaterial Delivery Systems Targeting TAMs for Immune-Mechanical Landscape Reshaping

Therapeutic Cargo	Delivery Vehicle/Platform	Targeting & Engineering Strategy	Therapeutic Outcomes & Mechanobiological Readouts	Ref.
BLZ945 (CSF-1R inhibitor)	Hydroxyl-terminated PAMAM dendrimers (D-BLZ)	Selective Intracellular Delivery: Leveraging nanoscale dimensions and hydrophilic shells for preferential accumulation within glioblastoma TAMs.	Achieved sustained intracellular release; significantly downregulated M2 markers, enhanced CD8⁺ T cell infiltration, and prolonged survival.	325
PLX3397 (CSF-1R inhibitor)	Nebulized/Localized pulmonary delivery system	Regional Spatial Targeting: Overcoming the pulmonary epithelial barrier via aerosolization to minimize systemic exposure and off-target toxicity.	Potently suppressed tumor burden at ultra-low doses (1 mg/kg); reduced M2-TAM density and enhanced M1-like antigen-presenting capacity.	326
anti-CSF-1R siRNA	anti-CSF-1R siRNA	Dual-Ligand Recognition: Surface-functionalized with SR-B1 targeting peptides and M2pep for high-fidelity identification of M2-like TAMs.	Induced a ~52% reduction in M2-TAMs and an 87% decrease in tumor volume; downregulated IL-10/TGF-β while promoting effector T cell responses.	327
Poly(I:C) + R848 (TLR7/8 agonists)	Mannose-hyaluronic acid coated polymer nanocapsules	Receptor-Mediated Endocytosis: Utilizing mannose-functionalized surfaces to target CD206⁺ TAMs for specific cellular internalization.	Triggered a robust M2-to-M1 phenotypic switch (CD86↑/CD206↓); suppressed primary tumor growth and distal metastasis in lung cancer models.	328
Chloroquine (CQ) + Polysaccharides	Redox-responsive PLGA nanoparticles	Metabolic-Immune Reprogramming: Exploiting the natural affinity of polysaccharides for pro-tumorigenic macrophages to co-deliver metabolic modulators.	Shifted TAM metabolic profiles from OXPHOS toward glycolysis; elevated M1-like polarization and augmented antitumor efficacy in breast cancer.	329
R848 (TLR7/8 agonist)	Cross-linked carboxymethyl dextran nanoparticles (Macrins)	Intrinsic Phagocytic Preference: Leveraging the inherent avidity of macrophages for dextran-based architectures to ensure targeted delivery.	Markedly upregulated IL-12 expression in TAMs; effectively inhibited tumor progression in MC38 colorectal cancer models.	217
R848 (TME-responsive NP)	M0 Macrophages as cell carriers (PR-M)	Cell-Mediated "Trojan Horse": Utilizing the innate tumor-homing capacity of macrophages for deep tissue penetration and targeted cargo release.	Achieved a 5.5-fold increase in M1/M2 ratio; robustly activated CD4⁺/CD8⁺ T cells and reduced tumor volume by 87.6%.	330
Doxorubicin (DOX)	Acid-sensitive PEGylated and mannose-modified PLGA NPs	Environment-Triggered Exposure: PEG shedding in the acidic TME to reveal mannose ligands for CD206-targeted uptake.	Enhanced TAM-specific internalization while minimizing hepatic and splenic sequestration; improved intratumoral drug distribution.	331
Sialic acid / Drug	Sialic acid-grafted liposomes	Siglec-axis targeting: Exploiting sialic acid affinity for circulating monocytes and TAMs.	Attenuates monocyte recruitment; slows M2-driven tissue remodeling.	306
IRF5 / IKKβ mRNA	Ligand-modified nanoparticles	Transcriptional rewiring: Encapsulating informational mRNAs to reprogram TAM signaling networks.	Drives stable M1 polarization; restrains collagen deposition and stiffening.	312
CRISPR/Cas9 (Pik3cg)	CpG-rich bacterial nanovesicles	Gene-level intervention: Co-delivery of Cas9 and TLR9 agonists for metabolic blockade.	Blocks PI3Kγ signaling; converts "cold" tumors to ICI-responsive states.	316
miR-125b	HA-PEI nanoparticles	CD44-mediated uptake: Utilizing hyaluronic acid (HA) for targeted miRNA delivery.	Induces robust M1-TAM conversion; potently inhibits metastatic progression.	321
siYTHDF1 (RNA methylation regulator)	RGD/mannose dual-targeted chromium nanoparticles	Multimodal Surface Engineering: Combining dual-receptor targeting with photothermal therapy (PTT)-triggered siRNA release.	Modulated the STAT3/STAT1 signaling axis; reshaped the myeloid landscape by reducing Treg and M2-TAM frequencies while enriching CD8⁺ T cells.	332

## Abbreviations

ADCs: Antibody-drug conjugates
α-SMA: Alpha-smooth muscle actin
ARG1: Arginase 1
CAFs: Cancer-associated fibroblasts
CAR-M: Chimeric antigen receptor macrophages
cGAS: Cyclic GMP-AMP synthase
CTGF: Connective tissue growth factor
CTLA-4: Cytotoxic T-lymphocyte-associated protein 4
CYR61: Cysteine-rich angiogenic inducer 61
DAMPs: Damage-associated molecular patterns
ECM: Extracellular matrix
FAK: Focal adhesion kinase
FAO: Fatty acid oxidation
HDAC: Histone deacetylase
HIF-1⍺: Hypoxia-inducible factor 1 alpha
ICD: Immunogenic cell death
ICIs: Immune checkpoint inhibitors
IDO: Indoleamine 2,3-dioxygenase
IFP: Interstitial fluid pressure
IL-10: Interleukin 10
LOX: Lysyl oxidase
LOXL2: Lysyl oxidase-like 2
MAPK: Mitogen-activated protein kinase
MDSCs: Myeloid-derived suppressor cells
MMPs: Matrix metalloproteinases
MT1-MMP: Membrane-type 1 matrix metalloproteinase (MMP-14)
myCAFs: Myofibroblast-like cancer-associated fibroblasts
OXPHOS: Oxidative phosphorylation
PD-1: Programmed cell death protein 1
PD-L1: Programmed death-ligand 1
PDAC: Pancreatic ductal adenocarcinoma
PDGF: Platelet-derived growth factor
PI3Kγ: Phosphoinositide 3-kinase gamma
PPARδ: Peroxisome proliferator-activated receptor delta
PYK2: Proline-rich tyrosine kinase 2
ROCK: Rho-associated coiled-coil-containing protein kinase
STING: Stimulator of interferon genes
TAMs: Tumor-associated macrophages
TAZ: Transcriptional coactivator with PDZ-binding motif
TBK1: TANK-binding kinase 1
TGF-β: Transforming growth factor beta
TLR: Toll-like receptor
TME: Tumor microenvironment
Tregs: Regulatory T cells
VEGF: Vascular endothelial growth factor
YAP: Yes-associated protein
